# Comparative genomics and mutagenesis analyses of choline metabolism in the marine *R* *oseobacter* clade

**DOI:** 10.1111/1462-2920.12943

**Published:** 2015-08-04

**Authors:** Ian Lidbury, George Kimberley, David J. Scanlan, J. Colin Murrell, Yin Chen

**Affiliations:** ^1^ School of Life SciencesUniversity of WarwickCoventryCV4 7ALUK; ^2^ School of Environmental SciencesUniversity of East AngliaNorwichNR4 7TJUK

## Abstract

Choline is ubiquitous in marine eukaryotes and appears to be widely distributed in surface marine waters; however, its metabolism by marine bacteria is poorly understood. Here, using comparative genomics and molecular genetic approaches, we reveal that the capacity for choline catabolism is widespread in marine heterotrophs of the marine *Roseobacter* clade (MRC). Using the model bacterium *R* *uegeria pomeroyi*, we confirm that the *bet* *A*, *bet* *B* and *bet* *C* genes, encoding choline dehydrogenase, betaine aldehyde dehydrogenase and choline sulfatase, respectively, are involved in choline metabolism. The *bet* *T* gene, encoding an organic solute transporter, was essential for the rapid uptake of choline but not glycine betaine (GBT). Growth of choline and GBT as a sole carbon source resulted in the re‐mineralization of these nitrogen‐rich compounds into ammonium. Oxidation of the methyl groups from choline requires formyltetrahydrofolate synthetase encoded by *fhs* in *R* *. pomeroyi*, deletion of which resulted in incomplete degradation of GBT. We demonstrate that this was due to an imbalance in the supply of reducing equivalents required for choline catabolism, which can be alleviated by the addition of formate. Together, our results demonstrate that choline metabolism is ubiquitous in the MRC and reveal the role of Fhs in methyl group oxidation in *R* *. pomeroyi*.

## Introduction

Choline is an essential constituent of eukaryotic cells where it can either be incorporated into the polar head group of the phospholipid phosphatidylcholine or sphingolipids (Ohvo‐Rekilä *et al*., 2002; Li and Vance, 2008). In mammals, choline plays an essential role in the transfer of methyl groups between cellular compounds and can be transformed into the neurotransmitter, acetylcholine (Ikawa and Taylor, 1973; Ueland, 2011). Choline also occurs in marine microalgae, e.g. in diatoms (Ikawa and Taylor, 1973), and a variety of coastal plants in the form of choline O‐sulfate (COS) (Catalfomo *et al*., 1972; Hanson and Gage, 1991; Hanson *et al*., 1991; 1994), and is a known osmoprotectant used by bacteria (Cánovas *et al*., 1996; Nau‐Wagner *et al*., 1999) and plants (Hanson and Gage, 1991; Hanson *et al*., 1991). Choline can be liberated from phosphatidylcholine through the action of phosphodiesterases which are present in the majority of plants, as well as viruses, bacteria, fungi and animals (Jenkins and Frohman, 2005). Due to its widespread occurrence in marine eukaryotes, choline appears to be ubiquitous in the marine water column, being detected in regions ranging from productive coastal waters of the English Channel to the oligotrophic North Atlantic gyre (Roulier *et al*., 1990; Airs and Archer, 2010).

It is known that marine bacteria can rapidly acquire choline from seawater (Kiene, 1998; Kiene *et al*., 1998) with the standing concentrations of choline being in the low nM range (Roulier *et al*., 1990; Airs and Archer, 2010). Choline, through its conversion to glycine betaine (GBT), serves as a potent osmoprotectant (Landfald and Strøm, 1986; Styrvold *et al*., 1986; Graham and Wilkinson, 1992; Boch *et al*., 1994). It is known, for example, that certain *Vibrio* spp. can convert choline to GBT to facilitate their survival in saline environments when they are not in association with their chosen hosts (Kapfthammer *et al*., 2005). In addition to being the precursor for the osmoprotectant GBT, choline is also a nutrient for bacteria. However, its catabolism in marine surface waters is not well understood (Kiene, 1998). In many bacteria, such as *Sinorhizobium meliloti* and *Pseudomonas aeruginosa*, catabolism of choline provides a growth advantage when forming close associations with their eukaryotic hosts (Smith *et al*., 1988; Barra *et al*., 2006; Sun *et al*., 2014).

The marine *Roseobacter* clade (MRC) are a monophyletic group of *Alphaproteobacteria* that are frequently detected during eukaryotic phytoplankton blooms and are often found in close association with a range of eukaryotic biota (González *et al*., 2000; Buchan *et al*., 2005; Porsby *et al*., 2008; Hahnke *et al*., 2013). These associations can change from a beneficial to an antagonistic relationship depending on the physiological state of either the host or the bacterium (Seyedsayamdost *et al*., 2011). Due to their high level of metabolic diversity and high *in situ* metabolic activity, the MRC plays a major role in carbon, sulfur and nitrogen cycling within dynamic coastal surface waters (González *et al*., 2000; Buchan *et al*., 2005; Moran and Miller, 2007; Chen, 2012; Lidbury *et al*., 2014b). MRC bacteria are also known for their competitive success (probiotic effect) against a number of marine‐associated pathogens through the production of antagonistic secondary metabolites (Porsby *et al*., 2008; Prado *et al*., 2009). It is, therefore, likely that MRC bacteria are capable of utilizing choline as an essential nutrient. Indeed, it has been reported that *Phaeobacter gallaeciensis* 2.10 and *Phaeobacter gallaeciensis* BS107, two isolates from the MRC, show weak growth on choline and its downstream metabolite, GBT (Thole *et al*., 2012). However, a comprehensive study of choline metabolism by members of the MRC has not been conducted.

Recent studies have revealed that methylated compounds, such as GBT (a metabolite of choline metabolism), methanol, dimethylsulfoniopropionate (DMSP) and trimethylamine (TMA), can be oxidized by members of the MRC and SAR11 clade to augment their growth on other organic substrates, and to maintain cell viability during times of carbon starvation through the generation of reducing equivalents and ATP (Sun *et al*., 2011; Lidbury *et al*., 2014b). SAR11 clade bacteria can also grow on GBT as a sole carbon source through its sequential demethylation to glycine and then pyruvate (Sun *et al*., 2011; Carini *et al*., 2013). Marine bacteria, including representatives from the MRC and SAR11 clade, lack the genes required for the oxidation of C1 groups to CO_2_ via the cofactor, tetrahydromethanopterin (H_4_MPT). It has, therefore, been proposed that in these marine heterotrophic bacteria, oxidation of the methyl groups from these compounds requires tetrahydrofolate as the cofactor (Chistoserdova, 2011; Sun *et al*., 2011; Chen, 2012; Lidbury *et al*., 2014b), involving several key enzymes, including formyltetrahydrofolate synthetase (Fhs). However, this has yet to be experimentally validated. Using comparative genomics and mutagenesis approaches, here, we studied choline metabolism in the MRC clade and investigated the role of Fhs in methyl group oxidation during choline metabolism using the model MRC bacterium *Ruegeria pomeroyi*.

## Results

### Choline and COS catabolism to GBT in *R* *. pomeroyi* requires three genes encoded by *betABC*

**Figure 1:**
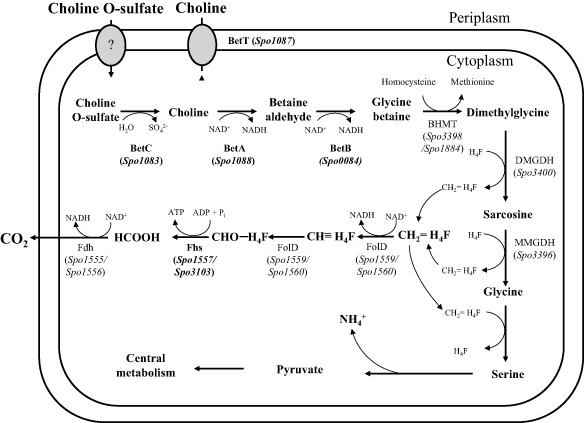
Proposed pathway of choline catabolism in *R* *uegeria pomeroyi* DSS‐3 and other related marine *R* *oseobacter* clade bacteria. HCOOH, formate; CHO‐H_4_F, formyl‐tetrahydrofolate; CHO = H_4_F, 5, 10‐methylene‐tetrahydrofolate; CHOΞH_4_F, 5, 10‐methenyl‐tetrahydrofolate; H_4_F, tetrahydrofolate; BetA, choline dehydrogenase; BetB, betaine aldehyde dehydrogenase; BetC, choline sulfatase; BetT, choline transporter; BHMT, glycine betaine: homocysteine methyltransferase; DMGDH, dimethylglycine dehydrogenase; MMGDH, sarcosine dehydrogenase; Fdh, formate dehydrogenase; FolD, 5,10‐methylene‐H_4_F dehydrogenase/ methenyl‐H_4_F cyclohydrolase; Fhs, formyl‐H_4_F synthetase; CO_2_, carbon dioxide.

Figure 1 shows the proposed pathway for the catabolism of choline and its metabolites in the model marine bacterium, *R. pomeroyi*. Enzymes required for the conversion of choline and COS to GBT (encoded by the *betA*, *betB* and *betC* genes) were identified in *R. pomeroyi* using blastp analysis, and they had 69%, 70% and 75% amino acid identity, respectively, to the characterized enzymes from the terrestrial bacterium *S. meliloti* (Smith *et al*., 1988; Østerås *et al*., 1998; Barra *et al*., 2006).

**Figure 2:**
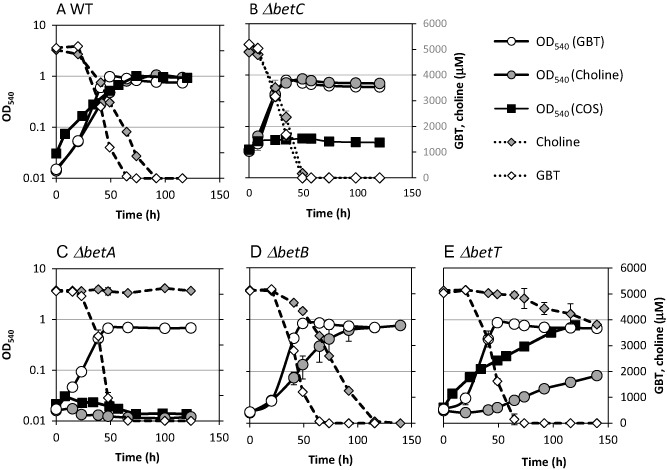
Growth of *R* *uegeria pomeroyi* (A) wild‐type (WT), (B) *bet* *C* mutant, (C) *bet* *A* mutant, (D) *bet* *B* mutant and (E) *bet* *T* mutant on either choline (grey circles), GBT (white circles) or COS (black squares) as the sole carbon source. Concentrations of choline (grey diamonds) and GBT (white diamonds) were quantified throughout the experiment. Cultures were grown in triplicate. Error bars denote standard deviations. GBT, glycine betaine; COS, choline O‐sulfate.

To confirm that *betABC* is essential for growth on choline and COS in *R. pomeroyi*, mutants of *betA*, *betB* and *betC* were constructed. Wild‐type *R. pomeroyi* grew well on choline (μ = 0.073 ± 0.003 h^−1^) and GBT (μ = 0.0860 ± 0.001 h^−1^), and a total of 5 mM GBT or choline was completely depleted from the culture medium after 72 and 95 h respectively (Fig. 2A). Growth on COS was slightly slower (μ = 0.043 ± 0.000 h^−1^). However, the final OD_540_ was comparable to that of choline and GBT (Fig. 2A). For the mutant strains, *ΔbetA::Gm*, *ΔbetB::Gm*, growth on GBT was not affected, while growth on choline was either completely or partially inhibited. Thus, the *ΔbetA::Gm* mutant failed to grow on choline as a sole carbon source, and no depletion of choline in the medium was observed (Fig. 2C). The *ΔbetB::Gm* (Fig. 2D) mutant could grow on choline as a sole carbon source; however, the growth rate was reduced (μ = 0.047 ± 0.008 h^−1^) compared with that of the wild‐type. During growth experiments on choline with *ΔbetB::Gm*, a transient build‐up of betaine aldehyde was detected in the culture medium, which was not evident in wild‐type cultures (data not shown). *R. pomeroyi* has a number of genes that may encode an aldehyde dehydrogenase similar to BetB, and it is likely that one of these enzymes was able to perform the same function as BetB, albeit at a reduced efficiency. As expected, the *ΔbetC::Gm* mutant could still utilize choline and GBT as a sole carbon source (Fig. 2B). However, this mutant strain failed to utilize COS as a carbon source, confirming that *betC* is essential for growth on COS.

### BetT is required for the uptake of, and growth on, choline in *R* *. pomeroyi*

In *R. pomeroyi*, directly upstream of *betA* is a putative *betT* gene (SPO1087), encoding a betaine‐carnitine‐choline transporter (BCCT), which is known to be responsible for the uptake of extracellular choline in *Escherichia coli* (Lamark *et al*., 1996). To investigate the role of *betT* in choline metabolism in *R. pomeroyi*, a *ΔbetT::Gm* mutant was constructed. The mutant could grow on choline as a sole carbon and energy source; however, the growth rate (μ = 0.012 ± 0.002 h^−1^) was severely reduced compared with that of the wild‐type (μ = 0.073 ± 0.003 h^−1^). Consequentially, the rate of choline depletion was also severely reduced (84%) compared with that of the wild‐type (Fig. 2E). Growth of the *ΔbetT::Gm* mutant on COS (μ = 0.027 ± 0.001 h^−1^) was also affected (wild‐type μ = 0.043 ± 0.000 h^−1^), showing a 38% reduction in growth rate.

### Degradation of choline and GBT by *R* *. pomeroyi* releases ammonium

**Figure 3:**
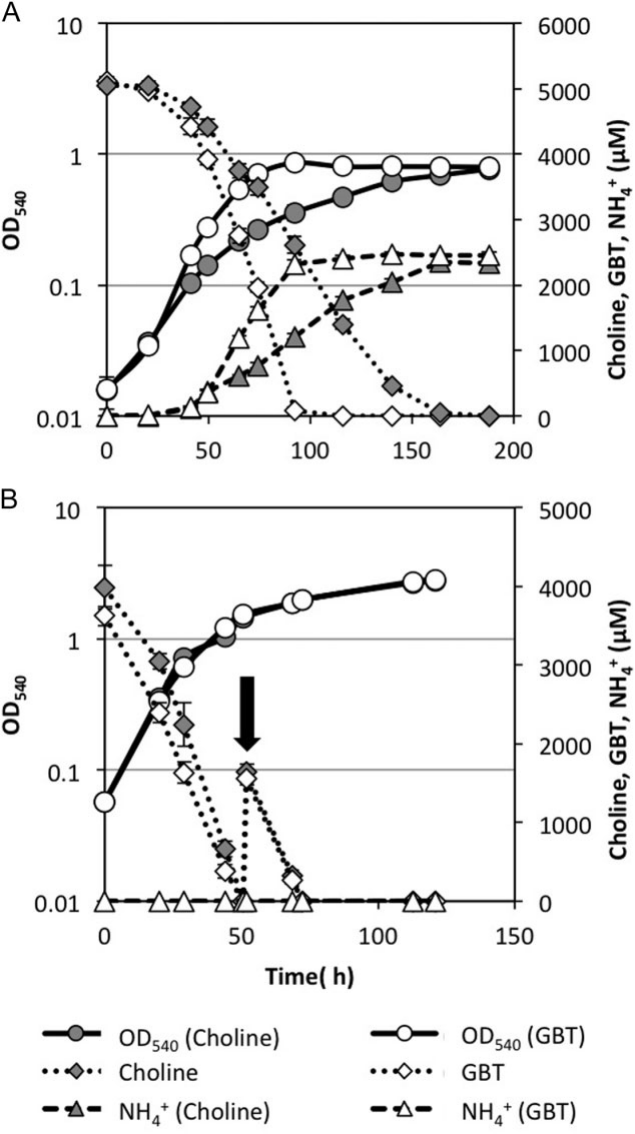
Growth of *R* *. pomeroyi* on choline (grey circles) or GBT (white circles) as a sole carbon and nitrogen source (5 mM) (A) or on choline or GBT (4 mM) as the sole nitrogen source with glucose (10 mM) added to the medium (B). Concentrations of choline (grey diamonds) and GBT (white diamonds) were quantified throughout the experiment. NH_4_ ^+^ was also quantified during the experiment in either GBT‐grown (white triangles) or choline‐grown cultures (grey triangles). Arrow indicates a second addition of either choline or GBT (∼ 2 mM). Cultures were grown in triplicate and error bars denote standard deviation. GBT, glycine betaine.

We showed previously that turnover of nitrogen‐rich methylated amines by marine bacteria, primarily as a source of supplementary energy, resulted in the remineralization of organic nitrogen in the form of ammonium (Lidbury *et al*., 2014b). Similarly, when either choline or GBT was used as the sole carbon and nitrogen source for *R. pomeroyi*, ammonium accumulation in the culture medium was observed (Fig. 3A). Moreover, the addition of glucose to the medium (increasing the carbon : nitrogen ratio above cell stoichiometry and thus making nitrogen the limiting nutrient) resulted in no accumulation of ammonium in the culture medium, despite the degradation of choline or GBT (Fig. 3B).

### *betABC* and choline transporters are widely distributed in marine bacteria of the *A* *lphaproteobacteria*, the MRC clade and some *G* *ammaproteobacteria*

**Figure 4:**
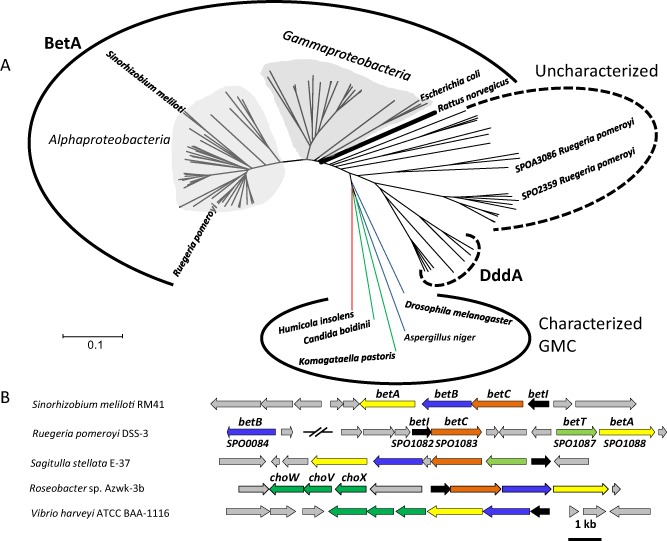
Phylogenetic analysis of choline dehydrogenase (BetA) in relation to other oxidoreductases of the glucose‐methanol‐choline (GMC) oxidoreductases family using the neighbour‐joining method (A). Bootstrap values from 500 replications were omitted for clarity. The tree was constructed using mega 5.2 (Tamura *et al*., 2011). A number of putative GMC oxidoreductases that are closely related to BetA were identified in marine heterotrophs, which have no assigned function, including two from *R* *. pomeroyi*. The blue lines denote glucose dehydrogenases, the red line denotes a cellobiose dehydrogenase, and the green lines represent methanol/alcohol dehydrogenases. (B) The genetic neighbourhood of the *bet* genes in representative marine bacterial isolates. DddA, 3‐hydroxypropionate dehydrogenase; BetA, choline dehydrogenase; *cho* *X*, periplasmic binding protein of the choline ABC transporter; *cho* *V*, ATP‐binding domain of the choline ABC transporter; *cho* *W*, transmembrane permease of the choline ABC transporter; *bet* *I*, regulator of *bet* operon; *bet* *T*, choline permease; *bet* *A*, choline dehydrogenase; *bet* *B*, betaine aldehyde dehydrogenase; *bet* *C*, choline sulfatase.

To better understand the potential importance of choline metabolism in the MRC, the genome sequences of isolates from the MRC were screened for the presence of the *betABC* genes required for choline metabolism. Out of 52 MRC genomes, 51 have the *betA* gene in their genomes, while 48 and 37 contain *betC* and *betB* respectively (Table 1). It is interesting that some strains lack *betB* as this gene was clearly involved but not essential for growth on choline in *R. pomeroyi* (Fig. 2). In the majority of isolates from the MRC, the *betABC* genes, together with the regulator *betI*, are found in a regulon, for example in isolates *Roseobacter* sp. Azwk‐3b and *Sagittula stellata* E‐37 (Fig. 4). Fourteen MRC isolates were screened for their ability to grow on choline and GBT as a sole carbon and energy source (Table 1). Growth on choline and COS as a sole carbon and energy source directly correlated with the presence of the *betABC* genes in their genomes. The genetic potential to use choline appears to be more widespread within the MRC than their ability to utilize methylamines, such as TMA (Chen *et al*., 2011) or monomethylamine (MMA) (Chen, 2012).

**Table 1:**
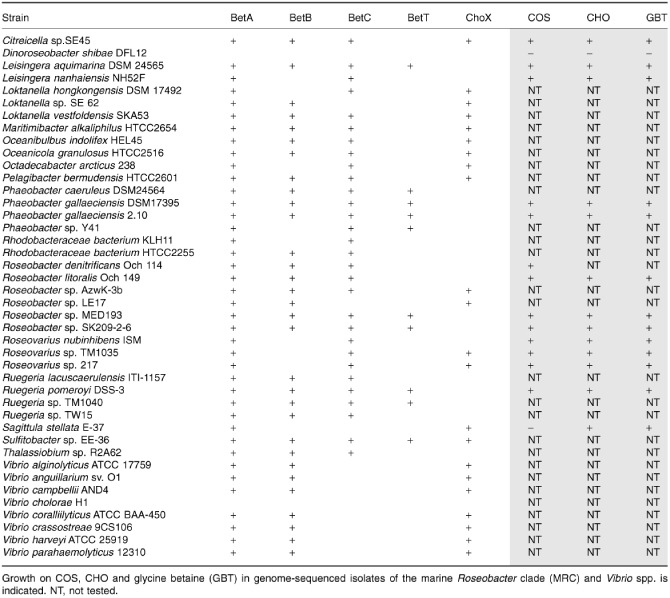
Comparative genomic analysis of genes involved in the catabolism of choline (CHO) and choline O‐sulfate (COS)

To better understand the distribution of choline metabolism genes among marine heterotrophs, we used BetA from *R. pomeroyi* as the query sequence to perform a blastp alignment scrutinizing the genomes of marine heterotrophs deposited in the Integrated Microbial Genomes database (http://img.jgi.doe.gov/). BetA belongs to the glucose‐methanol‐choline (GMC) oxidoreductase family (Cavener, 1992), including the characterized 3‐hydroxypropionate dehydrogenase (DddA) which is involved in DMSP catabolism (Curson *et al*., 2011). In addition to the MRC clade, BetA is also found in many isolates from the *Gammaproteobacteria*, including *Vibrio* spp. and *Alteromonas* spp. (Fig. 4). BetA homologues were also present in a number of single‐cell amplified genomes from abundant bacteria of the *Alphaproteobacteria* and *Gammaproteobacteria*, which were retrieved from marine surface waters (Swan *et al*., 2013) (Fig. 4, Fig. S3)*.* While representatives from the MRC possessed the *betC* gene required for COS degradation to choline, as well as genes required for the further catabolism of GBT to glycine (Table 1), representatives of *Vibrio* spp. did not. Furthermore, no BetA homologues were retrieved from the genomes of SAR11 clade bacteria.

The BCCT‐type choline transporter BetT is not present in all MRC bacteria (Fig. 4, Table 1). Instead, some MRC bacteria (e.g. *Roseovarius* sp. 217, *Octadecabacter arcticus* 238) have three open reading frames (ORFs) immediately upstream of the *betIABC* genes, which are annotated as genes encoding three subunits of an ABC‐type choline transporter (ChoXWV) (Chen *et al*., 2010). Phylogenetic analysis of the substrate‐binding protein, ChoX, from MRC bacteria reveals a close relationship with the ChoX from *S. meliloti* (Fig. S2; Chen *et al*., 2010), suggesting that this gene is likely to be involved in choline metabolism. The BetT‐type and the ChoX‐type choline transporters seem mutually exclusive in almost all MRC bacteria isolates (Table 1). The presence of the BetT‐type transporter is associated with MRC subclades one and two as defined by Newton and colleagues (2010), while the ABC‐type choline transporter, ChoXWV, is associated with MRC subclades three and four and *Vibrio* spp.

### The role of formyl tetrahydrofolate synthetase (Fhs) during choline metabolism in *R* *. pomeroyi*

In *R. pomeroyi*, complete degradation of choline to pyruvate results in the release of ammonium (Figs 1 and 3), while two of the three methyl groups arising from choline degradation are hypothesized to be conjugated to the carrier tetrahydrofolate (H_4_F) and further oxidized (Fig. 1). The other methyl group is predicted to be oxidized and conjugated to homocysteine, producing methionine (Fig. 1). Indeed, H_4_F‐binding domains were found in several key enzymes involved in choline catabolism, including dimethylglycine (DMG) dehydrogenase (SPO3400) and sarcosine dehydrogenase (SPO3396). It was hypothesized that complete oxidation of 5, 10‐methylenetetrahydrofolate (CH_2_ = H_4_F) through formyltetrahydrofolate synthetase (Fhs) provides reducing power in the form of NADH and ATP (Sun *et al*., 2011; Lidbury *et al*., 2014b). *R. pomeroyi* has two nearly identical copies (99.3% identity in nucleotide sequence, 100% identical in amino acid sequence) of the *fhs* gene (Fig. S4).

**Figure 5:**
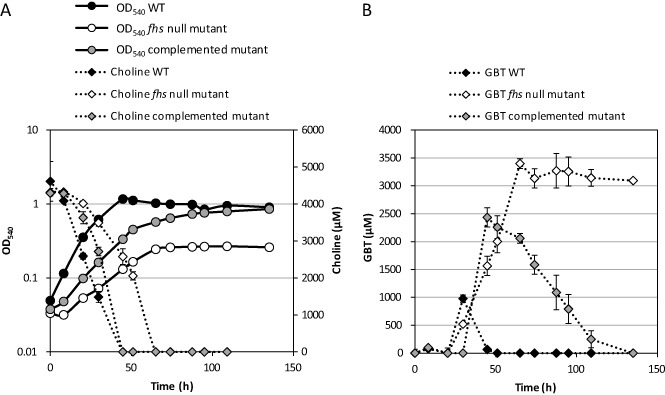
Growth (circles) of *R* *. pomeroyi* wild‐type, *fhs* null mutant and the complemented mutant on choline (5 mM) (diamonds) as a sole carbon, nitrogen and energy source (A). GBT (diamonds) in the culture medium was quantified throughout growth (B). Cultures were grown in triplicate. Error bars denote standard deviations.

To determine the role of *fhs* in the oxidation of methyl groups in *R. pomeroyi*, both copies of *fhs* in this bacterium were deleted, generating the double mutant, *Δfhs‐1::Gm/Δfhs‐2::Spc* (hereafter refer to as the *fhs* null mutant). Compared with the growth rate of the wild‐type on choline (μ = 0.073 ± 0.003 h^−1^), the *fhs* null mutant had a significantly reduced growth rate (μ = 0.033 ± 0.008 h^−1^) as well as a reduced final growth yield (*fhs* null mutant OD_540_ = 0.27, wild‐type OD_540_ = 1.17) (Fig. 5A). In cultures of the *fhs* null mutant, the initial rate of choline depletion was slower than that of the wild‐type; however, complete degradation of choline still occurred. During the experiment, there was a gradual build‐up of the metabolite GBT in cultures of the *fhs* null mutant (Fig. 5B). However, in wild‐type cultures, only a transient spike in GBT was observed. The complemented *fhs* mutant had a partially restored growth rate (μ = 0.050 ± 0.002 h^−1^) and final growth yield (OD_540_ = 0.84) (Fig. 5A) due to the restored ability to utilize GBT (Fig. 5B).

**Figure 6:**
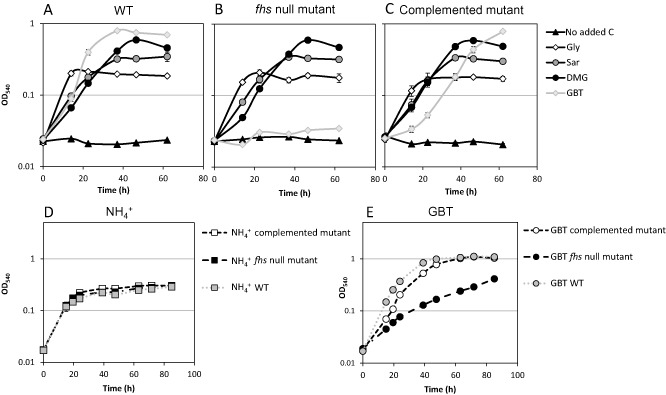
Growth of *R* *. pomeroyi* wild‐type (A), the *fhs* null mutant (B) and the complemented mutant (C) on 4 mM glycine (Gly), sarcosine (Sar), dimethylglycine (DMG) or glycine betaine (GBT) as the sole carbon source and ammonium (5 mM) as the nitrogen source. Negative growth control was set up without added carbon source. These three strains were also grown on 0.5 mM ammonium (NH_4_^+^) (D) or GBT (E) as a sole nitrogen source with glucose as the carbon source (10 mM). Cultures were grown in triplicate. Error bars denote standard deviations.

To determine whether the *fhs* mutation also affected growth on other downstream metabolites of choline metabolism, the wild‐type, the *fhs* null mutant and the complemented mutant were all grown on either glycine, sarcosine, DMG or GBT as a sole carbon and energy source. For the wild‐type, the final OD_540_ of the cultures showed a positive correlation with the increasing number of methyl groups, with growth on glycine resulting in the lowest OD_540_ and growth on GBT resulting in the highest yield (Fig. 6A). In the *fhs* null mutant, a similar phenotype was observed, but the null mutant failed to grow on GBT (Fig. 6B). Growth on GBT was, however, restored in the complemented mutant (Fig. 6C).

**Figure 7:**
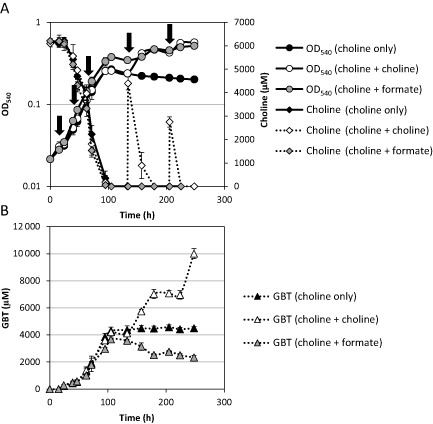
(A) Growth of the *fhs* null mutant (circles) on choline supplemented with either formate (grey circles) or additional choline (white circles). Choline was quantified throughout the experiment. Arrows denote additions of formate (13 mM total). The first three additions were with 1 mM formate, the latter two with 5 mM. (B) Quantification of GBT in the culture medium during the experiment. All cultures were grown in triplicate, and error bars denote standard deviations.

Although the null mutant cannot grow on GBT as a sole carbon source, we noticed that it could still grow on GBT as a nitrogen source, suggesting that sequential demethylation of GBT was still occurring in the null mutant, liberating ammonium as the source of nitrogen (Figs 1 and 6E). This suggests that the inability to demethylate GBT as the sole carbon source in the *fhs* null mutant was not due to a lack of recycled H_4_F. Indeed, experiments supplementing the *fhs* null mutant with either homocysteine or H_4_F failed to restore growth on GBT as the sole carbon source (Figs S5 and S6). Therefore, we hypothesized that GBT catabolism cannot function without Fhs due to an imbalance in the reducing state of the cell, i.e. the cell is limited by either the production of reducing equivalents and/or ATP. To test this hypothesis, the *fhs* null mutant was grown on choline and supplemented with formate to provide NADH through formate dehydrogenase (Fig. 1). The *fhs* null mutant grown in the presence of choline‐only reached a final OD_540_ ∼ 0.27, whereas supplementation with formate (13 mM total) resulted in almost double the amount of growth (OD_540_ ∼ 0.52) (Fig. 7A). Consequently, the concentration of GBT in cultures supplemented with formate was significantly reduced (Fig. 7B). The addition of more choline (∼ 8 mM) to half the choline‐only cultures resulted in the continuation of growth after cultures had reached stationary phase; meanwhile, a stepwise build‐up of GBT was also observed (Fig. 7B). Together, these data show that in *R. pomeroyi*, the reducing equivalents, as well as the ATP generated through oxidation of the C1 groups, are essential for maintaining adequate reducing power during choline, and specifically GBT catabolism.

## Discussion

In this study, the key genes responsible for the metabolism and subsequent growth on choline by members of the MRC have been experimentally confirmed. Marine eukaryotic flora accumulate COS as an osmolyte (Hanson and Gage, 1991; Hanson *et al*., 1991; Murakeözy *et al*., 2003), and the work in this study has confirmed that isolates from the MRC can utilize COS as a nutrient, using BetC. Phosphatidylcholine often accounts for > 50% of the phospholipid pool in certain eukaryotic fauna and flora (van Meer *et al*., 2008); therefore, phosphatidylcholine may provide a significant source of choline in niches associated with eukaryotic biota. The ability to metabolize choline and COS is ubiquitous in the MRC, and we speculate that these compounds may be an important nutrient source for these bacteria that are known to form close associations with eukaryotic biota (Ikawa and Taylor, 1973; González *et al*., 2000; Hjelm *et al*., 2004; Buchan *et al*., 2005; Porsby *et al*., 2008; Lema *et al*., 2014).

Bacteria related to the SAR11 clade can catabolize GBT, and homologues of the key enzymes involved in GBT metabolism, betaine homocysteine methyltransferase, DMG dehydrogenase and sarcosine dehydrogenase, have been identified in their genomes (Sun *et al*., 2011). However, to date, there is no physiological evidence to suggest that choline is a nutrient source for this clade. Furthermore, the genes involved in the uptake of choline (*betT*, *choX*) and subsequent catabolism (*betABC*) are absent from their genomes. It was previously shown that choline can be rapidly taken up by coastal marine bacteria and transformed to GBT (Gauthier and Le Rudulier, 1990; Ghoul *et al*., 1990; Kiene, 1998), and different phytoplankton species can also synthesize and/or acquire extracellular GBT to aid in osmoregulation (Keller *et al*., 1999; 2004). Consequentially, the concentration of particulate GBT is significantly higher than that of particulate choline in marine surface waters (Airs and Archer, 2010). In addition, a proportion of intracellular GBT can also be released back into the marine environment, through both passive and active mechanisms (Kapfthammer *et al*., 2005). Together, these data suggest that GBT is likely to be more widespread within the water column compared with choline, which may be a nutrient more commonly associated with niches surrounding eukaryotic biota. In support of this hypothesis, all characterized choline‐specific BCCT‐type transporters have only been identified in bacteria that form close associations with either a plant or animal host (Andresen *et al*., 1988; Fan *et al*., 2003; Chen and Beattie, 2008). Unlike many MRC bacteria, SAR11 bacteria are free‐living, oligotrophic cells that are not typically associated with eukaryotic flora or fauna (Morris *et al*., 2002; Giovannoni *et al*., 2005; Luo *et al*., 2013). The contrasting ability of choline and GBT catabolism in the MRC and the SAR11 clade bacteria may, therefore, reflect their different lifestyles and thus ecological niche separation.

The BCCT‐type transporter, BetT, found in the genomes of *R. pomeroyi* and other MRC bacteria appears different from the previous BetT choline transporters characterized from either *E. coli* or *P. syringae* in that those BetT proteins are over 100 amino acids longer (Chen and Beattie, 2008). In the same study, it was experimentally confirmed that the presence of an elongated C‐terminus is required for the uptake of choline under hyperosmotic stress. The authors proposed that the addition of an elongated C‐terminus could be used to predict the function of BetT, where the presence of an elongated C‐terminus denotes a role in osmoregulation and its absence denotes a role in the uptake of choline as a nutrient source (Chen and Beattie, 2008). Therefore, BetT in the MRC may primarily have a role in the uptake of choline as a nutrient. The *ΔbetT::Gm* mutant of *R. pomeroyi* showed no change in growth on GBT, suggesting that it is not a GBT transporter. This is in line with the fact that the majority of BCCT‐type transporters are characterized by having a narrow substrate range (Choquet *et al*., 2005; Chen and Beattie, 2008). The GBT transporter in *R. pomeroyi* awaits further experimental validation, and there are a number of potential candidates within its genome. We observed that the *ΔbetT::Gm* mutant showed reduced growth rates on choline and COS. This suggests that there is another transport system in place for the uptake of these compounds, similar to that of *Bacillus subtilis* (Nau‐Wagner *et al*., 2012).

The catabolism of nitrogen‐rich compounds provides a route for the remineralization of organic nitrogen back into ammonium, which can stimulate the growth of another bacterium in co‐culture (Lidbury *et al*., 2014b). Here, we provide further evidence that growth of marine heterotrophs on choline or GBT as a sole carbon source can also result in the remineralization of ammonium. Bacterioplankton in the Sargasso Sea, a region typified by prolonged periods of phosphate limitation and not nitrogen limitation (Wu *et al*., 2000), may well release ammonium during the oxidation of different nitrogen‐rich methylated compounds (Sun *et al*., 2011). The notion that nitrogen‐rich methylated compounds are primarily oxidized for carbon or energy is in line with the observation that phytoplankton seston is rapidly degraded by the bacterioplankton resulting in an increase of inorganic nitrogen, in the form of ammonium (Garber, 1984).

In *R. pomeroyi* and other marine heterotrophs, Fhs (encoded by *fhs*), which is involved in H_4_F‐mediated oxidation of methyl groups, is predicted to convert formyl‐H_4_F to formate, which can then be oxidized to CO_2_ (Chen, 2012). Fhs, therefore, plays an essential role not only in the recycling of H_4_F, making it available for further methyl group acceptance, but also providing ATP and reducing equivalents in the form of NADH resulting from the further catabolism of formate (Fig. 1) (Chistoserdova *et al*., 2004; Sun *et al*., 2011; Chen, 2012). Our experiments show that the turnover of GBT is affected in the *fhs* null mutant during growth, particularly when this compound represents the only source of ATP and reducing equivalents for the cell. However, our experiments do not clarify whether or not complete oxidation of the methyl groups to CO_2_ has been terminated. *R. pomeroyi* does possess the genes required for C1 oxidation through the glutathione‐linked (GSH) C1 oxidation pathway, which has previously been shown to alleviate stress caused by formaldehyde toxicity (Harms *et al*., 1996; Marx *et al*., 2003; Martinez‐Gomez *et al*., 2013). The GSH‐linked pathway usually requires the enzyme formaldehyde‐activating enzyme (Fae) to facilitate the conjugation of formaldehyde to GSH or to the alternative cofactor H_4_MPT (increasing the rate of conjugation by up to 10‐fold) (Goenrich *et al*., 2002; Chen, 2012). Unlike the majority of non‐marine representative methylotrophs, isolates from the MRC, including *R. pomeroyi*, lack *fae*. Therefore, it is unclear whether or not the GSH‐linked pathway can deal with any potential build‐up of formaldehyde during the catabolism of choline and GBT in the *fhs* null mutant. In *Methylobacterium extorquens* PA1, formaldehyde leakage via the gamma‐glutamylmethylamide/*N*‐methylglutamate pathway was observed during growth on MMA (Nayak and Marx, 2014). Therefore, in the *fhs* null mutant, formaldehyde leakage, due to a potential lack of free H_4_F, may present a problem for the cell and may explain the slower growth rates observed for the *fhs* null mutant when growing on GBT as a sole nitrogen source. In reality, a combination of impaired reducing power generation and free H_4_F is the likely explanation behind the phenotypes observed in the *fhs* null mutant. This was supported by the fact that the *fhs* null mutant failed to grow on GBT as a sole carbon source without the addition of another source of reducing power, such as formate.

In summary, we demonstrate that the ability to utilize choline is a universal trait of MRC bacteria which requires the enzymes BetABC. Based on comparative genomic analyses, choline metabolism appears to be absent in SAR11 clade bacteria. In addition, our study has also confirmed the hypothesis that the H_4_F‐linked C1 oxidation pathway has a role in the oxidation of the methyl groups released during the degradation of methylated compounds, which is required to maintain normal cell physiology.

## Experimental procedures

### Cultivation of bacteria

The MRC isolates were maintained on marine agar 2216 (Difco, UK) or ½ YPSS: yeast extract (2 g l^−1^), peptone (1.25 g l^−1^) and sea salts (30 g l^−1^, Sigma). Gentamicin (10 μg ml^−1^), kanamycin (80 μg ml^−1^) or spectinomycin (175 μg ml^−1^) was added to maintain *R. pomeroyi* mutant strains, *ΔbetA::Gm*, *ΔbetB::Gm*, *ΔbetC::Gm*, *ΔbetT::Gm*, *Δfhs‐1::Gm/Δfhs‐2::Spc*, and the complemented mutant strain, *Δfhs‐1::GmΔ/fhs‐2::Spc* + *fhs‐1:DSS‐3*. Choline O‐sulfate was purchased from the Cambridge Isotope Laboratories. For all growth experiments, *R. pomeroyi* (wild‐type and mutants) as well as other strains from the MRC were grown in a marine ammonium mineral salts (MAMS) medium with the addition of relevant carbon sources. The MAMS medium as modified by Schäfer (2007) contained the following (per litre): NaCl, 20 g; (NH_4_)_2_SO_4,_ 1 g; MgSO_4_·7H_2_O, 1 g; CaCl_2_·2H_2_O, 0.2 g; FeSO_4_·7H_2_O, 2 mg; Na_2_MoO_4_·2H_2_O, 20 mg; KH_2_PO_4_, 0.36 g; K_2_HPO_4_, 2.34 g; plus 1 ml of SL‐10 trace metals solution (Schäfer, 2007). Vitamins were prepared as described previously (Chen, 2012). To determine if choline and GBT were used as a nitrogen source and whether growth on choline or GBT led to the release of ammonium, (NH_4_)_2_SO_4_ was removed from the standard MAMS recipe, and either GBT or choline was added to the medium (at concentrations of 4 or 5 mM) with 10 mM glucose.

### Genetic manipulation of *R* *. pomeroyi*

A full list of strain and plasmids used in this study is outlined in Table 2. To construct mutants in *R. pomeroyi*, two regions of genomic DNA were amplified, one towards the 5′ end (with restriction sites engineered at either end) and the other towards the 3′ end (with restriction sites engineered at each end) of the target genes. Sequence integrity after subcloning into the pGEM‐T vector was confirmed via DNA sequencing. The complete list of primers used, and restriction sites introduced to generate the mutants used in this study, is shown in Table S1. An upstream and downstream fragment of the target gene, along with either the gentamicin gene cassette, amplified from p34S‐Gm (Dennis and Zylstra, 1998), or the spectinomycin cassette, amplified from pHP45Ω (Prentki and Krisch, 1984), were subcloned into the cloning vector pGEM‐T (Promega). The entire construct was then excised from pGEM‐T and ligated into the suicide vector pK18mob*sacB* (Schäfer *et al*., 1994). The resulting plasmid was transformed into *Escherichia coli* S17.1 via electroporation and mobilized into *R. pomeroyi* via conjugation onto a 0.22 μm pore‐size, 47 mm sterile filter (Millipore, UK), using 1/2 YTSS (Deutsche Sammlung von Mikroorganismen und Zellkulturen, DSMZ) as the medium. Transconjugants were selected for on the sea salts minimal medium as described previously with gentamicin (10 μg ml^−1^) and MMA (3 mM) as a sole nitrogen source (Lidbury *et al*., 2014a). Double cross‐over mutants were selected by their sensitivity to kanamycin, and homologous recombination was confirmed by polymerase chain reaction (PCR) and DNA sequencing.

**Table 2:** List of strains and plasmids used in this study

Strains/plasmids	Description/use	Source
*E. coli* S17.1	Electrocompetent cells used for conjugation	Lab collection
*E. coli* JM109	Routine host for cloning	Promega
*R. pomeroyi* DSS‐3	Wild‐type	González and colleagues (2003)
*R. pomeroyi* Δ*betA*::Gm	*R. pomeroyi* with disrupted *betA*	This study
*R. pomeroyi* Δ*betB*::Gm	*R. pomeroyi* with disrupted *betB*	This study
*R. pomeroyi* Δ*betC*::Gm	*R. pomeroyi* with disrupted *betC*	This study
*R. pomeroyi* Δ*betT*::Gm	*R. pomeroyi* with disrupted *betT*	This study
*R. pomeroyi* Δ*fhs‐1*::Gm Δfhs‐2::Spc	*R. pomeroyi* with both copies of fhs disrupted	This study
*R. pomeroyi* Δ*fhs* + *fhs*:DSS‐3	*fhs* null mutant complemented with native *fhs‐1*	This study
p34S‐Gm	Source of a gentamicin gene cassette	Dennis and Zylstra (1998)
pK18mob*sacB*	Suicide vector for *R. pomeroyi* (Kan^R^)	Schäfer and colleagues (1994)
pBBR1MCS‐km	Broad‐host‐range plasmid (Kan^R^)	Kovach and colleagues (1995)
pHP45Ω	Source of spectinomycin gene cassette	Prentki and Krisch (1984)
pKIL301	Mutated *betA* and the gentamicin cassette cloned into pK18mob*sacB*	This study
pKIL302	Mutated *betB* and the gentamicin cassette cloned into pK18mob*sacB*	This study
pKIL303	Mutated *betC* and the gentamicin cassette cloned into pK18mob*sacB*	This study
pKIL304	Mutated *betT* and the gentamicin cassette cloned into pK18mob*sacB*	This study
pKIL305	Mutated *fhs‐1* and the gentamicin cassette cloned into pK18mob*sacB*	This study
pKIL306	Mutated *fhs‐2* and the spectinomycin cassette cloned into pK18mob*sacB*	This study
pBIL301	Native *fhs* and the promoter upstream on the *fhs‐1* operon cloned into the vector pBBR1MCS‐km	This study

To complement the *Δfhs‐1::GmΔfhs‐2::Spc* mutant, the *fhs‐1* gene (encoded by SPO1557), and the native promoter for the operon were amplified and individually subcloned into pGEM‐T. The promoter and *fhs‐1* were sequentially cloned into the broad‐host range plasmid, pBBR1MCS‐km (Kovach *et al*., 1995) using the restriction sites *Kpn*I and *Sal*I, and *Sal*I and *Bam*HI respectively. The resultant plasmid, pBIL105, was mobilized into the *R. pomeroyi* mutant as described above. Confirmation of the complemented mutant was carried out by PCR and DNA sequencing.

### Quantification of quaternary and methylated amines

Cells were removed from culture medium by centrifugation (10 000 × *g*, 2 min) of spin columns (0.22 μm pore‐size, nylon, Costar, Corning, NY). All amines and ammonium, apart from COS, were quantified using a cation‐exchange ion chromatograph equipped with a Metrosep C4/250‐mm separation column and a conductivity detector (Metrohm) as described previously (Lidbury *et al*., 2014a).

### Comparative genomic analysis of the genes involved in the metabolism of choline and related compounds

For all analyses, the Integrated Microbial Genomes database at the Joint Genome Institute (IMG/JGI) was used to identify all genes involved in the metabolism of quaternary amines. Each blast search was conducted using an E‐value of 1e‐20 with a minimum sequence identity cut‐off of 30%. Searches were conducted using all marine heterotrophic bacteria in the IMG/JGI database selecting genomes with draft/finished/permanent draft status, after which representative sequences from different bacterial clades/groups were selected. The locus tag and accession numbers (Gene ID) for the following genes used as query sequences were as follows: *betA*, SMc00093, 637181738; *betB*, SMc00094, 637181739; *betC*, SMc00127, 637181740; *betT*, 637288573; *choX*, 638910580. Phylogenetic analysis was conducted using the mega 5.2 package (Tamura *et al*., 2011). The National Centre for Biotechnology Information database was used to find a number of sequences not in the IMG/JGI database. Due to the high number of hits retrieved, a stringent E‐value < 1e‐170 was used to identify true homologues. Where appropriate, phylogenetic analysis was performed to infer function.

## Supporting information


**Fig. S1.** Phylogenetic analysis of the substrate binding proteins (SBPs) affiliated with the putative choline ABC‐type transporter found in marine bacteria. Reference sequences from characterized SBPs were added to the alignment. Characterized SBPs, related to osmolyte SBPs, based on the structural analysis conducted by Berntsson and colleagues (2010) were used as an outgroup. The tree was aligned in mega 5.2 using the neighbour‐joining method using 500 replications for bootstrapping. The scale bar represents the number of substitutions per amino acid. ChoX, SBP specific for choline; TmoX, SBP specific for trimethylamine *N*‐oxide; BetX, SBP specific for glycine betaine; CaiX, SBP specific for carnitine.
**Fig. S2.** Detailed phylogeny of ChoX from Fig. S1 showing strain names and their corresponding accession numbers (Gene ID in IMG/JGI).
**Fig. S3.** Phylogenetic analysis of choline dehydrogenase (BetA). The evolutionary history was inferred using the neighbour‐joining method. For the major nodes, the percentage (> 75%) of replicate trees in which the associated taxa clustered together in the bootstrap test (500 replicates) are shown. The tree is drawn to scale, with branch lengths in the same units as those of the evolutionary distances used to infer the phylogenetic tree. The evolutionary distances were computed using the p‐distance method and are in the units of the number of amino acid differences per site. The analysis involved 101 amino acid sequences. All ambiguous positions were removed for each sequence pair. There were a total of 685 positions in the final dataset. Evolutionary analyses were conducted in mega6.
**Fig. S4.** Gene neighbourhoods of *fhs1* and *fhs2*. The scale bar represents the number of bases. *folD*, 5,10‐methylene‐H_4_F dehydrogenase/methenyl‐H_4_F cyclohydrolase; *tmm*, trimethylamine monooxygenase; *tdm*, trimethylamine *N*‐oxide demethylase; *fhs*, formyl‐H_4_F synthetase; *tmoR*, putative regulator of *tmm*; *amt*, unspecified ammonium transporter; *ftsH*, ATP‐dependent metalloprotease; *fhdA,* formate dehydrogenase *alpha* subunit; *fhdB*, formate dehydrogenase *beta* subunit; PBP, uncharacterized HAAT family amino acid periplasmic binding protein.
**Fig. S5.** Growth of the *R. pomeroyi fhs* null mutant on GBT (red squares), homocysteine (purple crosses) or GBT and homocysteine (green triangles) as the carbon source respectively. A positive control consisted of glucose as a carbon source and the negative control had no added carbon. Cultures were grown in triplicate. Error bars denote SD. Hcy, homocysteine.
**Fig. S6.** Growth of *R. pomeroyi* wild‐type and the *fhs* null mutant on glucose and GBT as the carbon and energy source and ammonium as the nitrogen source. Tetrahydrofolate (1 mM) was added to wild‐type and mutant cultures at T = 0 h and T = 21 h, and GBT consumption was recorded. Cultures were grown in triplicate. Error bars denote SD.
**Table S1.** List of oligonucleotides used in this study.

## References

[emi12943-bib-0001] Airs, R.L. , and Archer, S.D. (2010) Analysis of glycine betaine and choline in seawater particulates by liquid chromatography/electrospray ionization/mass spectrometry. Limnol Oceanog‐Meth 8: 499–506.

[emi12943-bib-0002] Andresen, P.A. , Kaasen, I. , Styrvold, O.B. , Boulnois, G. , and Strom, A.R. (1988) Molecular cloning, physical mapping and expression of the bet genes governing the osmoregulatory choline‐glycine betaine pathway of *Escherichia coli* . J Gen Microbiol 134: 1737–1746. 3065456 10.1099/00221287-134-6-1737

[emi12943-bib-0003] Barra, L. , Fontenelle, C. , Ermel, G. , Trautwetter, A. , Walker, G.C. , and Blanco, C. (2006) Interrelations between glycine betaine catabolism and methionine biosynthesis in *Sinorhizobium meliloti* strain 102F34. J Bacteriol 188: 7195–7204. 17015658 10.1128/JB.00208-06 PMC1636217

[emi12943-bib-0004] Boch, J. , Kempf, B. , and Bremer, E. (1994) Osmoregulation in *Bacillus subtilis*: synthesis of the osmoprotectant glycine betaine from exogenously provided choline. J Bacteriol 176: 5364–5371. 8071213 10.1128/jb.176.17.5364-5371.1994 PMC196722

[emi12943-bib-0005] Buchan, A. , González, J.M. , and Moran, M.A. (2005) Overview of the marine *Roseobacter* lineage. Appl Environ Microbiol 71: 5665–5677. 16204474 10.1128/AEM.71.10.5665-5677.2005 PMC1265941

[emi12943-bib-0006] Cánovas, D. , Vargas, C. , Csonka, L.N. , Ventosa, A. , and Nieto, J.J. (1996) Osmoprotectants in *Halomonas elongata*: high‐affinity betaine transport system and choline‐betaine pathway. J Bacteriol 178: 7221–7226. 8955405 10.1128/jb.178.24.7221-7226.1996 PMC178636

[emi12943-bib-0007] Carini, P. , Steindler, L. , Beszteri, S. , and Giovannoni, S.J. (2013) Nutrient requirements for growth of the extreme oligotroph ‘*Candidatus* Pelagibacter ubique’ HTCC1062 on a defined medium. ISME J 7: 592–602. 23096402 10.1038/ismej.2012.122 PMC3578571

[emi12943-bib-0008] Catalfomo, P. , Block, J.H. , Constantine, G.H. , and Kirk, P.W. (1972) Choline sulfate (ester) in marine higher fungi. Mar Chem 1: 157–162.

[emi12943-bib-0009] Cavener, D.R. (1992) GMC oxidoreductases: a newly defined family of homologous proteins with diverse catalytic activities. J Mol Biol 223: 811–814. 1542121 10.1016/0022-2836(92)90992-s

[emi12943-bib-0010] Chen, C. , and Beattie, G.A. (2008) *Pseudomonas syringae* BetT is a low‐affinity choline transporter that is responsible for superior osmoprotection by choline over glycine betaine. J Bacteriol 190: 2717–2725. 18156257 10.1128/JB.01585-07 PMC2293270

[emi12943-bib-0011] Chen, C. , Malek, A.A. , Wargo, M.J. , Hogan, D.A. , and Beattie, G.A. (2010) The ATP‐binding cassette transporter Cbc (choline/betaine/carnitine) recruits multiple substrate‐binding proteins with strong specificity for distinct quaternary ammonium compounds. Mol Microbiol 75: 29–45. 19919675 10.1111/j.1365-2958.2009.06962.x PMC5503199

[emi12943-bib-0012] Chen, Y. (2012) Comparative genomics of methylated amine utilisation by marine *Roseobacter* clade bacteria and development of functional gene markers (*tmm*, *gmaS*). Environ Microbiol 14: 2308–2322. 22540311 10.1111/j.1462-2920.2012.02765.x

[emi12943-bib-0013] Chen, Y. , Patel, N.A. , Crombie, A. , Scrivens, J.H. , and Murrell, J.C. (2011) Bacterial flavin‐containing monooxygenase is trimethylamine monooxygenase. Proc Natl Acad Sci USA 108: 17791–17796. 22006322 10.1073/pnas.1112928108 PMC3203794

[emi12943-bib-0014] Chistoserdova, L. (2011) Modularity of methylotrophy, revisited. Environ Microbiol 13: 2603–2622. 21443740 10.1111/j.1462-2920.2011.02464.x

[emi12943-bib-0015] Chistoserdova, L. , Laukel, M. , Portais, J.‐C. , Vorholt, J.A. , and Lidstrom, M.E. (2004) Multiple formate dehydrogenase enzymes in the facultative methylotroph *Methylobacterium extorquens* AM1 are dispensable for growth on methanol. J Bacteriol 186: 22–28. 14679220 10.1128/JB.186.1.22-28.2004 PMC303455

[emi12943-bib-0016] Choquet, G. , Jehan, N. , Pissavin, C. , Blanco, C. , and Jebbar, M. (2005) OusB, a broad‐specificity ABC‐type transporter from *Erwinia chrysanthemi*, mediates uptake of glycine betaine and choline with a high affinity. Appl Environ Microbiol 71: 3389–3398. 16000740 10.1128/AEM.71.7.3389-3398.2005 PMC1169054

[emi12943-bib-0017] Curson, A.R.J. , Todd, J.D. , Sullivan, M.J. , and Johnston, A.W.B. (2011) Catabolism of dimethylsulfoniopropionate: microorganisms, enzymes and genes. Nat Rev Micro 9: 849–859. 10.1038/nrmicro2653 21986900

[emi12943-bib-0018] Dennis, J.J. , and Zylstra, G.J. (1998) Plasposons: modular self‐cloning minitransposon derivatives for rapid genetic analysis of gram‐negative bacterial genomes. Appl Environ Microbiol 64: 2710–2715. 9647854 10.1128/aem.64.7.2710-2715.1998 PMC106450

[emi12943-bib-0019] Fan, X. , Pericone, C.D. , Lysenko, E. , Goldfine, H. , and Weiser, J.N. (2003) Multiple mechanisms for choline transport and utilisation in *Haemophilus influenzae* . Mol Microbiol 50: 537–548. 14617177 10.1046/j.1365-2958.2003.03703.x

[emi12943-bib-0020] Garber, J.H. (1984) Laboratory study of nitrogen and phosphorus remineralization during the decomposition of coastal plankton and seston. Estuar Coast Shelf Sci 18: 685–702.

[emi12943-bib-0021] Gauthier, M.J. , and Le Rudulier, D. (1990) Survival in seawater of *Escherichia coli* cells grown in marine sediments containing glycine betaine. Appl Environ Microbiol 56: 2915–2918. 2275537 10.1128/aem.56.9.2915-2918.1990 PMC184864

[emi12943-bib-0022] Ghoul, M. , Bernard, T. , and Cormier, M. (1990) Evidence that *Escherichia coli* accumulates glycine betaine from marine sediments. Appl Environ Microbiol 56: 551–554. 2407188 10.1128/aem.56.2.551-554.1990 PMC183376

[emi12943-bib-0023] Giovannoni, S.J. , Tripp, H.J. , Givan, S. , Podar, M. , Vergin, K.L. , Baptista, D. , *et al* (2005) Genome streamlining in a cosmopolitan oceanic bacterium. Science 309: 1242–1245. 16109880 10.1126/science.1114057

[emi12943-bib-0024] Goenrich, M. , Bartoschek, S. , Hagemeier, C.H. , Griesinger, C. , and Vorholt, J.A. (2002) A glutathione‐dependent formaldehyde‐activating enzyme (Gfa) from *Paracoccus denitrificans* detected and purified via two‐dimensional proton exchange NMR spectroscopy. J Biol Chem 277: 3069–3072. 11741920 10.1074/jbc.C100579200

[emi12943-bib-0025] González, J.M. , Simó, R. , Massana, R. , Covert, J.S. , Casamayor, E.O. , Pedrós‐Alió, C. , and Moran, M.A. (2000) Bacterial community structure associated with a dimethylsulfoniopropionate‐producing North Atlantic algal bloom. Appl Environ Microbiol 66: 4237–4246. 11010865 10.1128/aem.66.10.4237-4246.2000 PMC92291

[emi12943-bib-0026] González, J.M. , Covert, J.S. , Whitman, W.B. , Henriksen, J.R. , Mayer, F. , Scharf, B. , *et al* (2003) *Silicibacter pomeroyi* sp. nov. and *Roseovarius nubinhibens* sp. nov., dimethylsulfoniopropionate‐demethylating bacteria from marine environments. Int J Syst Evol Micro 53: 1261–1269. 10.1099/ijs.0.02491-0 13130004

[emi12943-bib-0027] Graham, J.E. , and Wilkinson, B.J. (1992) *Staphylococcus aureus* osmoregulation: roles for choline, glycine betaine, proline, and taurine. J Bacteriol 174: 2711–2716. 1556089 10.1128/jb.174.8.2711-2716.1992 PMC205912

[emi12943-bib-0028] Hahnke, S. , Brock, N.L. , Zell, C. , Simon, M. , Dickschat, J.S. , and Brinkhoff, T. (2013) Physiological diversity of *Roseobacter* clade bacteria co‐occurring during a phytoplankton bloom in the North Sea. Syst Appl Microbiol 36: 39–48. 23265193 10.1016/j.syapm.2012.09.004

[emi12943-bib-0029] Hanson, A.D. , and Gage, D.A. (1991) Identification and determination by fast atom bombardment mass spectrometry of the compatible solute choline‐o‐sulfate in *Limonium* species and other halophytes. Aus J Plant Physiol 18: 317–327.

[emi12943-bib-0030] Hanson, A.D. , Rathinasabapathi, B. , Chamberlin, B. , and Gage, D.A. (1991) Comparative physiological evidence that β‐alanine betaine and choline‐O‐sulfate act as compatible osmolytes in halophytic *Limonium* species. Plant Physiol 97: 1199–1205. 16668509 10.1104/pp.97.3.1199 PMC1081142

[emi12943-bib-0031] Hanson, A.D. , Rathinasabapathi, B. , Rivoal, J. , Burnet, M. , Dillon, M.O. , and Gage, D.A. (1994) Osmoprotective compounds in the *Plumbaginaceae*: a natural experiment in metabolic engineering of stress tolerance. Proc Natl Acad Sci USA 91: 306–310. 8278383 10.1073/pnas.91.1.306 PMC42936

[emi12943-bib-0032] Harms, N. , Ras, J. , Reijnders, W.N. , van Spanning, R.J. , and Stouthamer, A.H. (1996) S‐formylglutathione hydrolase of *Paracoccus denitrificans* is homologous to human esterase D: a universal pathway for formaldehyde detoxification? J Bacteriol 178: 6296–6299. 8892832 10.1128/jb.178.21.6296-6299.1996 PMC178503

[emi12943-bib-0033] Hjelm, M. , Riaza, A. , Formoso, F. , Melchiorsen, J. , and Gram, L. (2004) Seasonal incidence of autochthonous antagonistic *Roseobacter* spp. and *Vibrionaceae* strains in a Turbot larva (*Scophthalmus maximus*) rearing system. Appl Environ Microbiol 70: 7288–7294. 15574928 10.1128/AEM.70.12.7288-7294.2004 PMC535194

[emi12943-bib-0034] Ikawa, M. , and Taylor, R.F. (1973) Choline and related substances in Algae In Marine Pharmacognosy: Action of Marine Biotoxins at the Cellular Level. Martin D., and Padilla G.M. (eds). New York, USA: Academic Press, pp. 203–236.

[emi12943-bib-0035] Jenkins, G.M. , and Frohman, M.A. (2005) Phospholipase D: a lipid centric review. Cell Mol Life Sci CMLS 62: 2305–2316. 16143829 10.1007/s00018-005-5195-z PMC11139095

[emi12943-bib-0036] Kapfthammer, D. , Karatan, E. , Pflughoeft, K.J. , and Watnick, P.I. (2005) Role for glycine betaine transport in Vibrio cholerae osmoadaptation and biofilm formation within microbial communities. Appl Environ Microbiol 71: 3840–3847. 16000796 10.1128/AEM.71.7.3840-3847.2005 PMC1169069

[emi12943-bib-0037] Keller, M.D. , Kiene, R.P. , Matrai, P.A. , and Bellows, W.K. (1999) Production of glycine betaine and dimethylsulfoniopropionate in marine phytoplankton. II. N‐limited chemostat cultures. Mar Biol 135: 249–257.

[emi12943-bib-0038] Keller, M.D. , Matrai, P.A. , Kiene, R.P. , and Bellows, W.K. (2004) Responses of coastal phytoplankton populations to nitrogen additions: dynamics of cell‐associated dimethylsulfoniopropionate (DMSP), glycine betaine (GBT), and homarine. Can J Fish Aquat Sci 61: 685–699.

[emi12943-bib-0039] Kiene, R.P. (1998) Uptake of choline and its conversion to glycine betaine by bacteria in estuarine waters. Appl Environ Microbiol 64: 1045–1051. 16349511 10.1128/aem.64.3.1045-1051.1998 PMC106365

[emi12943-bib-0040] Kiene, R.P. , Hoffmann Williams, L.P. , and Walker, J.E. (1998) Seawater microorganisms have a high affinity glycine betaine uptake system which also recognizes dimethylsulfoniopropionate. Aqua Microb Ecol 15: 39–51.

[emi12943-bib-0041] Kovach, M.E. , Elzer, P.H. , Steven Hill, D. , Robertson, G.T. , Farris, M.A. , Roop Ii, R.M. , and Peterson, K.M. (1995) Four new derivatives of the broad‐host‐range cloning vector pBBR1MCS, carrying different antibiotic‐resistance cassettes. Gene 166: 175–176. 8529885 10.1016/0378-1119(95)00584-1

[emi12943-bib-0042] Lamark, T. , Røkenes, T.P. , McDougall, J. , and Strøm, A.R. (1996) The complex bet promoters of *Escherichia coli*: regulation by oxygen (ArcA), choline (BetI), and osmotic stress. J Bacteriol 178: 1655–1662. 8626294 10.1128/jb.178.6.1655-1662.1996 PMC177851

[emi12943-bib-0043] Landfald, B. , and Strøm, A.R. (1986) Choline‐glycine betaine pathway confers a high level of osmotic tolerance in *Escherichia coli* . J Bacteriol 165: 849–855. 3512525 10.1128/jb.165.3.849-855.1986 PMC214506

[emi12943-bib-0044] Lema, K.A. , Bourne, D.G. , and Willis, B.L. (2014) Onset and establishment of diazotrophs and other bacterial associates in the early life history stages of the coral *Acropora millepora* . Mol Ecol 23: 4682–4695. 25156176 10.1111/mec.12899

[emi12943-bib-0045] Li, Z. , and Vance, D.E. (2008) Thematic review series: Glycerolipids. Phosphatidylcholine and choline homeostasis. J Lipid Res 49: 1187–1194. 18204095 10.1194/jlr.R700019-JLR200

[emi12943-bib-0046] Lidbury, I. , Murrell, J.C. , and Chen, Y. (2014a) Trimethylamine *N*‐oxide metabolism by abundant marine heterotrophic bacteria. Proc Natl Acad Sci USA 111: 2710–2715. 24550299 10.1073/pnas.1317834111 PMC3932874

[emi12943-bib-0047] Lidbury, I.D.E.A. , Murrell, J.C. , and Chen, Y. (2014b) Trimethylamine and trimethylamine *N*‐oxide are supplementary energy sources for a marine heterotrophic bacterium: implications for marine carbon and nitrogen cycling. ISME J 9: 760–769. 25148480 10.1038/ismej.2014.149 PMC4331573

[emi12943-bib-0048] Luo, H. , Csűros, M. , Hughes, A.L. , and Moran, M.A. (2013) Evolution of divergent life history strategies in marine *Alphaproteobacteria* . mBio 4: e00373‐13. 10.1128/mBio.00373-13 PMC3735120 23839216

[emi12943-bib-0049] Martinez‐Gomez, N.C. , Nguyen, S. , and Lidstrom, M.E. (2013) Elucidation of the role of the methylene‐tetrahydromethanopterin dehydrogenase MtdA in the tetrahydromethanopterin‐dependent oxidation pathway in *Methylobacterium extorquens* AM1. J Bacteriol 195: 2359–2367. 23504017 10.1128/JB.00029-13 PMC3650556

[emi12943-bib-0050] Marx, C.J. , Chistoserdova, L. , and Lidstrom, M.E. (2003) Formaldehyde‐detoxifying role of the tetrahydromethanopterin‐linked pathway in *Methylobacterium extorquens* AM1. J Bacteriol 185: 7160–7168. 14645276 10.1128/JB.185.23.7160-7168.2003 PMC296243

[emi12943-bib-0051] van Meer, G. , Voelker, D.R. , and Feigenson, G.W. (2008) Membrane lipids: where they are and how they behave. Nat Rev Mol Cell Biol 9: 112–124. 18216768 10.1038/nrm2330 PMC2642958

[emi12943-bib-0052] Moran, M.A. , and Miller, W.L. (2007) Resourceful heterotrophs make the most of light in the coastal ocean. Nat Rev Micro 5: 792–800. 10.1038/nrmicro1746 17828280

[emi12943-bib-0053] Morris, R.M. , Rappe, M.S. , Connon, S.A. , Vergin, K.L. , Siebold, W.A. , Carlson, C.A. , and Giovannoni, S.J. (2002) SAR11 clade dominates ocean surface bacterioplankton communities. Nature 420: 806–810. 12490947 10.1038/nature01240

[emi12943-bib-0054] Murakeözy, É.P. , Nagy, Z. , Duhazé, C. , Bouchereau, A. , and Tuba, Z. (2003) Seasonal changes in the levels of compatible osmolytes in three halophytic species of inland saline vegetation in Hungary. J Plant Physiol 160: 395–401. 12756919 10.1078/0176-1617-00790

[emi12943-bib-0055] Nau‐Wagner, G. , Boch, J. , Le Good, J.A. , and Bremer, E. (1999) High affinity transport of choline‐O‐sulfate and its use as a compatible solute in *Bacillus subtilis* . Appl Environ Microbiol 65: 560–568. 9925583 10.1128/aem.65.2.560-568.1999 PMC91062

[emi12943-bib-0056] Nau‐Wagner, G. , Opper, D. , Rolbetzki, A. , Boch, J. , Kempf, B. , Hoffmann, T. , and Bremer, E. (2012) Genetic control of osmoadaptive glycine betaine synthesis in *Bacillus subtilis* through the choline‐sensing and glycine betaine‐responsive GbsR repressor. J Bacteriol 194: 2703–2714. 22408163 10.1128/JB.06642-11 PMC3347207

[emi12943-bib-0057] Nayak, D.D. , and Marx, C.J. (2014) Methylamine utilization via the N‐methylglutamate pathway in *Methylobacterium extorquens* PA1 involves a novel flow of carbon through c1 assimilation and dissimilation pathways. J Bacteriol 196: 4130–4139. 25225269 10.1128/JB.02026-14 PMC4248863

[emi12943-bib-0058] Newton, R.J. , Griffin, L.E. , Bowles, K.M. , Meile, C. , Gifford, S. , Givens, C.E. , *et al* (2010) Genome characteristics of a generalist marine bacterial lineage. ISME J 4: 784–798. 20072162 10.1038/ismej.2009.150

[emi12943-bib-0059] Ohvo‐Rekilä, H. , Ramstedt, B. , Leppimäki, P. , and Slotte, J.P. (2002) Cholesterol interactions with phospholipids in membranes. Prog Lipid Res 41: 66–97. 11694269 10.1016/s0163-7827(01)00020-0

[emi12943-bib-0060] Østerås, M. , Boncompagni, E. , Vincent, N. , Poggi, M.‐C. , and Le Rudulier, D. (1998) Presence of a gene encoding choline sulfatase in *Sinorhizobium meliloti bet* operon: Choline‐O‐sulfate is metabolized into glycine betaine. Proc Natl Acad Sci USA 95: 11394–11399. 9736747 10.1073/pnas.95.19.11394 PMC21653

[emi12943-bib-0061] Porsby, C.H. , Nielsen, K.F. , and Gram, L. (2008) *Phaeobacter* and *Ruegeria* species of the *Roseobacter* clade colonize separate niches in a Danish Turbot (*Scophthalmus maximus*)‐rearing farm and antagonise *Vibrio anguillarum* under different growth conditions. Appl Environ Microbiol 74: 7356–7364. 18952864 10.1128/AEM.01738-08 PMC2592939

[emi12943-bib-0062] Prado, S. , Montes, J. , Romalde, J.L. , and Barja, J.L. (2009) Inhibitory activity of *Phaeobacter* strains against aquaculture pathogenic bacteria. Int Microbiol 12: 107–114. 19784930

[emi12943-bib-0063] Prentki, P. , and Krisch, H.M. (1984) In vitro insertional mutagenesis with a selectable DNA fragment. Gene 29: 303–313. 6237955 10.1016/0378-1119(84)90059-3

[emi12943-bib-0064] Roulier, M.A. , Palenik, B. , and Morel, F.M.M. (1990) A method for the measurement of choline and hydrogen peroxide in seawater. Mar Chem 30: 409–421.

[emi12943-bib-0065] Schäfer, A. , Tauch, A. , Jäger, W. , Kalinowski, J. , Thierbach, G. , and Pühler, A. (1994) Small mobilizable multi‐purpose cloning vectors derived from the *Escherichia coli* plasmids pK18 and pK19: selection of defined deletions in the chromosome of *Corynebacterium glutamicum* . Gene 145: 69–73. 8045426 10.1016/0378-1119(94)90324-7

[emi12943-bib-0066] Schäfer, H. (2007) Isolation of *Methylophaga* spp. from marine dimethylsulfide‐degrading enrichment cultures and identification of polypeptides induced during growth on dimethylsulfide. Appl Environ Microbiol 73: 2580–2591. 17322322 10.1128/AEM.02074-06 PMC1855583

[emi12943-bib-0067] Seyedsayamdost, M.R. , Case, R.J. , Kolter, R. , and Clardy, J. (2011) The Jekyll‐and‐Hyde chemistry of *Phaeobacter gallaeciensis* . Nat Chem 3: 331–335. 21430694 10.1038/nchem.1002 PMC3376411

[emi12943-bib-0068] Smith, L.T. , Pocard, J.A. , Bernard, T. , and Le Rudulier, D. (1988) Osmotic control of glycine betaine biosynthesis and degradation in *Rhizobium meliloti* . J Bacteriol 170: 3142–3149. 3290197 10.1128/jb.170.7.3142-3149.1988 PMC211261

[emi12943-bib-0069] Styrvold, O.B. , Falkenberg, P. , Landfald, B. , Eshoo, M.W. , Bjørnsen, T. , and Strøm, A.R. (1986) Selection, mapping, and characterization of osmoregulatory mutants of *Escherichia coli* blocked in the choline‐glycine betaine pathway. J Bacteriol 165: 856–863. 3512526 10.1128/jb.165.3.856-863.1986 PMC214507

[emi12943-bib-0070] Sun, J. , Steindler, L. , Thrash, J.C. , Halsey, K.H. , Smith, D.P. , Carter, A.E. , *et al* (2011) One carbon metabolism in SAR11 pelagic marine bacteria. PLoS ONE 6: e23973. 21886845 10.1371/journal.pone.0023973 PMC3160333

[emi12943-bib-0071] Sun, Z. , Kang, Y. , Norris, M.H. , Troyer, R.M. , Son, M.S. , Schweizer, H.P. , *et al* (2014) Blocking phosphatidylcholine utilisation in *Pseudomonas aeruginosa*, via mutagenesis of fatty acid, glycerol and choline degradation pathways, confirms the importance of this nutrient source *in vivo* . PLoS ONE 9: e103778. 25068317 10.1371/journal.pone.0103778 PMC4113454

[emi12943-bib-0072] Swan, B.K. , Tupper, B. , Sczyrba, A. , Lauro, F.M. , Martinez‐Garcia, M. , González J.M. *et al* (2013) Prevalent genome streamlining and latitudinal divergence of planktonic bacteria in the surface ocean. Proc Natl Acad Sci USA 110: 11463–11468. 23801761 10.1073/pnas.1304246110 PMC3710821

[emi12943-bib-0073] Tamura, K. , Peterson, D. , Peterson, N. , Stecher, G. , Nei, M. , and Kumar, S. (2011) MEGA5: molecular evolutionary genetics analysis using maximum likelihood, evolutionary distance, and maximum parsimony methods. Mol Biol Evol 28: 2731–2739. 21546353 10.1093/molbev/msr121 PMC3203626

[emi12943-bib-0074] Thole, S. , Kalhoefer, D. , Voget, S. , Berger, M. , Engelhardt, T. , Liesegang, H. , *et al* (2012) *Phaeobacter gallaeciensis* genomes from globally opposite locations reveal high similarity of adaptation to surface life. ISME J 6: 2229–2244. 22717884 10.1038/ismej.2012.62 PMC3504968

[emi12943-bib-0075] Ueland, P.M. (2011) Choline and betaine in health and disease. J Inherit Metab Dis 34: 3–15. 20446114 10.1007/s10545-010-9088-4

[emi12943-bib-0076] Wu, J. , Sunda, W. , Boyle, E.A. , and Karl, D.M. (2000) Phosphate depletion in the western North Atlantic Ocean. Science 289: 759–762. 10926534 10.1126/science.289.5480.759

