# Supplementary material for: Comparative genomics and mutagenesis analyses of choline metabolism in the marine R oseobacter clade
Source: Environ Microbiol. 2015 Aug 4;17(12):5048–62. doi: 10.1111/1462-2920.12943 (PMC4744692; doi:10.1111/1462-2920.12943)
Supplement: Supplementary file 1 — Fig. S1. Phylogenetic analysis of the substrate binding proteins (SBPs) affiliated with the putative choline ABC‐type transporter found in marine bacteria. Reference sequences from characterized SBPs were added to the alignment. Characterized SBPs, related to osmolyte SBPs, based on the structural analysis conducted by Berntsson and colleagues (2010) were used as an outgroup. The tree was aligned in mega 5.2 using the neighbour‐joining method using 500 replications for bootstrapping. The scale bar represents the number of substitutions per amino acid. ChoX, SBP specific for choline; TmoX, SBP specific for trimethylamine N‐oxide; BetX, SBP specific for glycine betaine; CaiX, SBP specific for carnitine. Fig. S2. Detailed phylogeny of ChoX from Fig. S1 showing strain names and their corresponding accession numbers (Gene ID in IMG/JGI). Fig. S3. Phylogenetic analysis of choline dehydrogenase (BetA). The evolutionary history was inferred using the neighbour‐joining method. For the major nodes, the percentage (> 75%) of replicate trees in which the associated taxa clustered together in the bootstrap test (500 replicates) are shown. The tree is drawn to scale, with branch lengths in the same units as those of the evolutionary distances used to infer the phylogenetic tree. The evolutionary distances were computed using the p‐distance method and are in the units of the number of amino acid differences per site. The analysis involved 101 amino acid sequences. All ambiguous positions were removed for each sequence pair. There were a total of 685 positions in the final dataset. Evolutionary analyses were conducted in mega6. Fig. S4. Gene neighbourhoods of fhs1 and fhs2. The scale bar represents the number of bases. folD, 5,10‐methylene‐H4F dehydrogenase/methenyl‐H4F cyclohydrolase; tmm, trimethylamine monooxygenase; tdm, trimethylamine N‐oxide demethylase; fhs, formyl‐H4F synthetase; tmoR, putative regulator of tmm; amt, unspecified ammonium transporter; ftsH, ATP‐dependent met [file EMI-17-5048-s001.docx]

**Supplementary information**

**Supplementary figure legends**

**Figure S1** Phylogenetic analysis of the substrate binding proteins (SBPs) affiliated with the putative choline ABC-type transporter found in marine bacteria. Reference sequences from characterised SBPs were added to the alignment. Characterised SBPs, related to osmolyte SBPs, based on the structural analysis conducted by Berntsson et al. (2010) were used as an outgroup. The tree was aligned in MEGA 5.2 using the neighbour-joining method using 500 replications for bootstrapping. The scale bar represents the number of substitutions per amino acid. Abbreviations: ChoX, SBP specific for choline; TmoX, SBP specific for trimethylamine *N*-oxide; BetX, SBP specific for glycine betaine; CaiX, SBP specific for carnitine.

**Figure S2** Detailed phylogeny of ChoX from Figure S1 showing strain names and their corresponding accession numbers (Gene ID in IMG/JGI).

**Figure S3** Phylogenetic analysis of choline dehydrogenase (BetA). The evolutionary history was inferred using the Neighbour-Joining method. For the major nodes, the percentage (>75%) of replicate trees in which the associated taxa clustered together in the bootstrap test (500 replicates) are shown. The tree is drawn to scale, with branch lengths in the same units as those of the evolutionary distances used to infer the phylogenetic tree. The evolutionary distances were computed using the p-distance method and are in the units of the number of amino acid differences per site. The analysis involved 101 amino acid sequences. All ambiguous positions were removed for each sequence pair. There were a total of 685 positions in the final dataset. Evolutionary analyses were conducted in MEGA6.

**Figure S4** Gene neighbourhoods of *fhs1* and *fhs2*. The scale bar represents the number of bases. Abbreviations: *folD*, 5,10-methylene-H_4_F dehydrogenase/ methenyl-H_4_F cyclohydrolase; *tmm*, trimethylamine monooxygenase; *tdm*, trimethylamine *N*-oxide demethylase; *fhs*, formyl-H_4_F synthetase; *tmoR*, putative regulator of *tmm*; *amt*, unspecified ammonium transporter; *ftsH*, ATP-dependent metalloprotease; *fhdA,* formate dehydrogenase *alpha* subunit; *fhdB*, formate dehydrogenase *beta* subunit; PBP, uncharacterised HAAT family amino acid periplasmic binding protein.

**Figure S5** Growth of the *R. pomeroyi fhs* null mutant on GBT (red squares), homocysteine (purple crosses) or GBT and homocysteine (green triangles) as the carbon source, respectively. A positive control consisted of glucose as a carbon source and the negative control had no added carbon. Cultures were grown in triplicate. Error bars denote s.d. Hcy: homocysteine.

**Figure S6** Growth of *R. pomeroyi* wild-type and the *fhs* null mutant on glucose and GBT as the carbon and energy source and ammonium as the nitrogen source. Tetrahydrofolate (1 mM) was added to wild-type and mutant cultures at T=0 h and T=21 h and GBT consumption was recorded. Cultures were grown in triplicate. Error bars denote s.d.

**Table S1**. List of oligonucleotides used in this study

| **Primer** | **Sequence** | **Use** |
| --- | --- | --- |
| Spo1088_AF_HindIII | CAATAAGCTTGGCAGCAAGGAAAGACAGAC | Cloning 5' end (region A) of *betA* |
| Spo1088_AR_BamHI | CAATGGATCCGCTTCTCGCCGTTATAGTCG | Cloning 5' end (region A) of *betA* |
| Spo1088_BF_BamHI | CAATGGATCCAATGGCTGTTCACCAAGACC | Cloning 3' end (region B) of *betA* |
| Spo1088_BR_XbaI | CAATTCTAGAATTGGTGATCTGCGGAAAGA | Cloning 3' end (region B) of *betA* |
| Spo1087_Cho_perm_AF_PstI | CAATCTGCAGGATGTCCCGAATGCGTTT | Cloning 5' end (region A) of *betT* |
| Spo1087_Cho_perm_AR_XbaI | CAATTCTAGATTGTTGACCAGCTTCACC AG | Cloning 5' end (region A) of *betT* |
| Spo1087_Cho_perm_BF_XbaI | CAATTCTAGACCTTGGCGATCTGGTTCTC | Cloning 3' end (region B) of *betT* |
| Spo1087_Cho_perm_BR_HindIII | CAATAAGCTTACGATGACAAAATCCGCT TC | Cloning 3' end (region B) of *betT* |
| Spo0084_AF_PstI | CAATCTGCAGATTTTCAACTCTGCCCGTTT | Cloning 5' end (region A) of *betB* |
| Spo0084_AR_XbaI | CAATTCTAGAACGGACGGTATAGGCGAAAT | Cloning 5' end (region A) of *betB* |
| Spo0084_BF_XbaI | CAATTCTAGAATATGTCGACAAGGGCAAGG | Cloning 3' end (region B) of *betB* |
| Spo0084_BR_HindIII | CAATAAGCTTATTCCGGCTTCTTTCTGGTT | Cloning 3' end (region B) of *betB* |
| Spo1083 _AF_PstI | CAATCTGCAGTCCTGATCCTGATGGTGGAT | Cloning 5' end (region A) of *betC* |
| Spo1083_ AR_BamHI | CAATGGATCCGATCGTAAAGCTTGGCCTTG | Cloning 5' end (region A) of *betC* |
| Spo1083_BF_BamHI | CAATGGATCCACACGCCGGTTTCTACCAT | Cloning 3' end (region B) of *betC* |
| Spo1083_BR_XbaI | CAATTCTAGACCTCTGGCTTTCCTCCAGAT | Cloning 3' end (region B) of *betC* |
| Spo1557_AF_XbaI: | TCTAGAGTCACCTATCCCTCGCTCAG | Cloning upstream of 5' end (region A) of *fhs1* |
| Spo1557_AR_SalI | ATGCTGTCGACTACGCCATCTGATGATTTCC | Cloning upstream of 5' end (region A) of *fhs1* |
| Spo1557_BF_SalI | ATGCTGTCGACGATCGAGGGCTTGTTCTGAG | Cloning downstream of 3' end (region B) of *fhs1* |
| Spo1557_BR_HindIII | AAGCTTTTCAGCTCTGCCACATGTTC | Cloning downstream of 3' end (region B) of *fhs1* |
| Spo3103_AF_ | CAATTCTAGAATTTCCATGCGATCACCAGC | Cloning 5' end (region A) of *fhs2* |
| Spo3103_AR_ | CCAATGGATCCACGTAATCGGCCACTTTCA | Cloning 5' end (region A) of *fhs2* |
| Spo3103_BF_ | CAATGGATCCGGCGATCAACCATTTCGTTCA | Cloning 3' end (region B) of *fhs2* |
| Spo3103_BR_ | CCAATAAGCTTCATTCAACCGGATGGTCTCT | Cloning 3' end (region B) of *fhs2* |
| Spo1088_CON_F1 | CTATATCGCGGGCAATGTCG | Confirmation of *ΔbetA::Gm* |
| Spo1088_CON_R1 | GACAGGGATCAAATCGGGTG | Confirmation of *ΔbetA::Gm* |
| Spo1087_CON_F1 | CATGCAGGATCGACAACAGG | Confirmation of *ΔbetT::Gm* |
| Spo1087_CON_R1 | GTTGTTCAGATGCGGTTCGG | Confirmation of *ΔbetT::Gm* |
| Spo0084_CON_F1 | GATACCGGTCGAAGGGAGAG | Confirmation of *ΔbetB::Gm* |
| Spo0084_CON_R1 | GGCAGGACAATCTTTCACGG | Confirmation of *ΔbetB::Gm* |
| Spo1083_CON_F1 | CTGATCGACGGGCTCTACAT | Confirmation of *ΔbetC::Gm* |
| Spo1083_CON_R1 | GCCATCACGTAGGTTTCGAC | Confirmation of *ΔbetC::Gm* |
| Spo1557_CON_F1 | GAGATGAAGCGCAACATGAA | Confirmation of *Δfhs1:Gm* |
| Spo1557_CON_R1 | TACCCAGAAGACCCACGTTC | Confirmation of *Δfhs1::Gm* |
| Spo3103_CON_F1 | GACCATCGACATGGAAAACC | Confirmation of *Δfhs2::Spc in Δfhs1::Gm* |
| Spo3103_CON_R1 | TTCAGCTCTGCCACATGTTC | Confirmation of *Δfhs2::Spc in Δfhs1::Gm* |
| Fhs_promF1_KpnI | CAATGGTACCTCTTGTGGGCCAA | Cloning the promoter for the T4F-kinked oxidation pathway |
| Fhs_promR1_SalI | CAATGTCGACCCGTCAACACCTC | Cloning the promoter for the T4F-kinked oxidation pathway |
| Fhs-1_F1_SalI | CAATGTCGACATGGCGTACAAGA | Cloning the *fhs* in *R. pomeroyi* |
| Fhs_1_R1_BamHI | CAATGGATCCTCAGAACAAGCCCTCGATCTG | Cloning the *fhs* in *R. pomeroyi* |

**Figure S1**

FIII cluster subgroups I, II, IV, V

BetX, CaiX and other non-characterised osmolyte SBPs found in marine bacteria

TmoX

ChoX


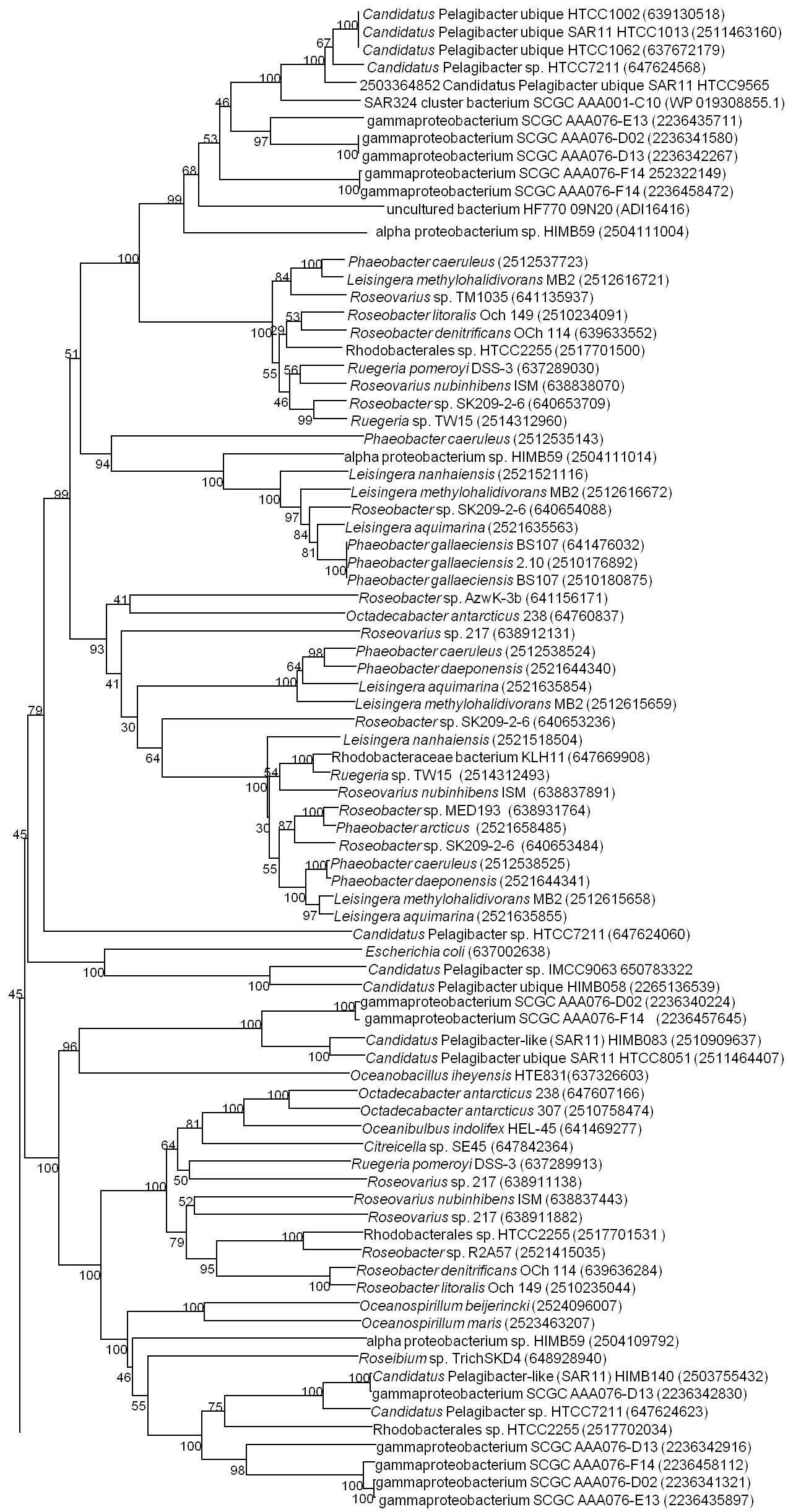


**Figure S2**

BetX

TmoX


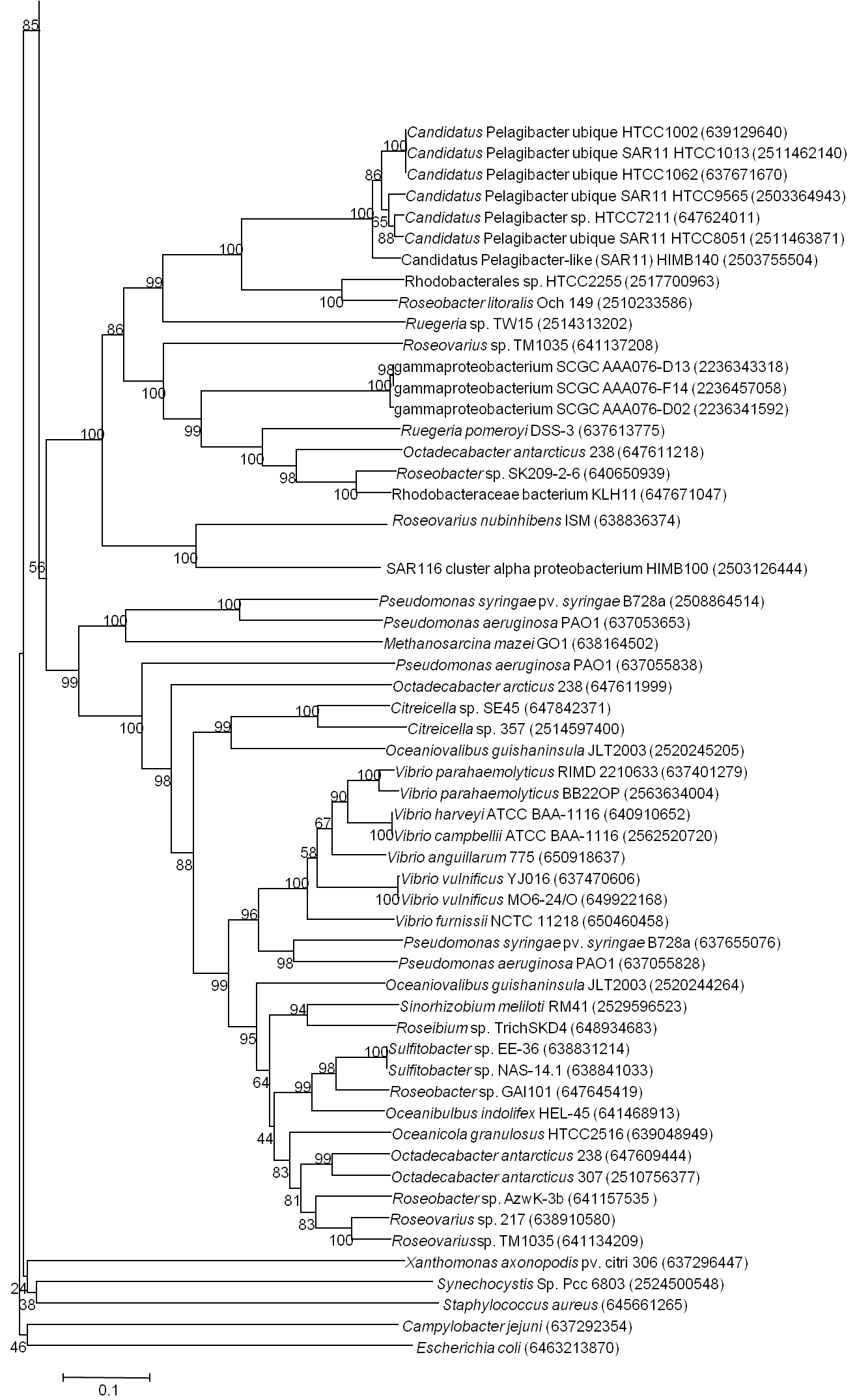


Cluster FIII

ChoX

CaiX

BetX

**
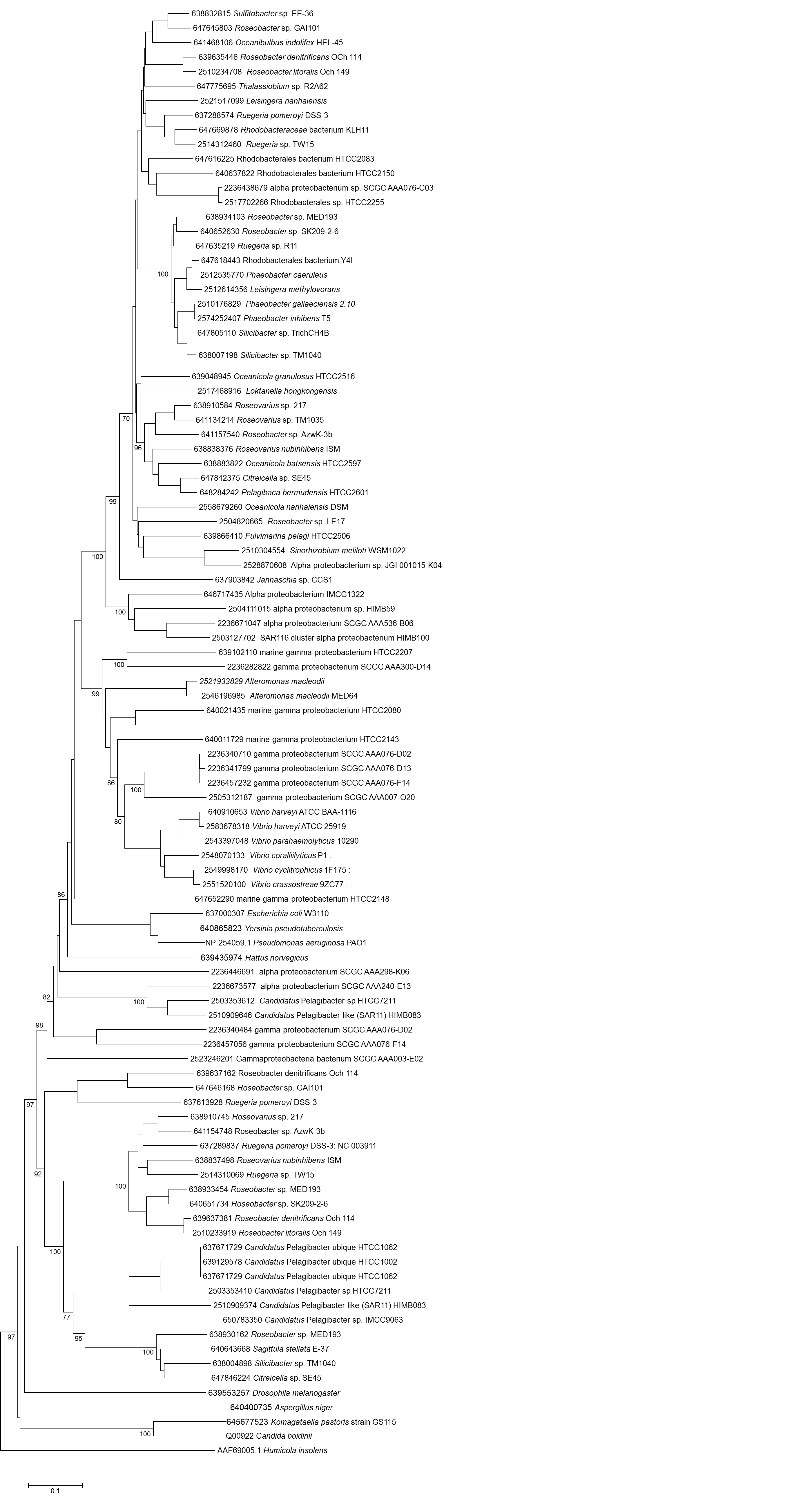
**

BetA

**Figure S3**


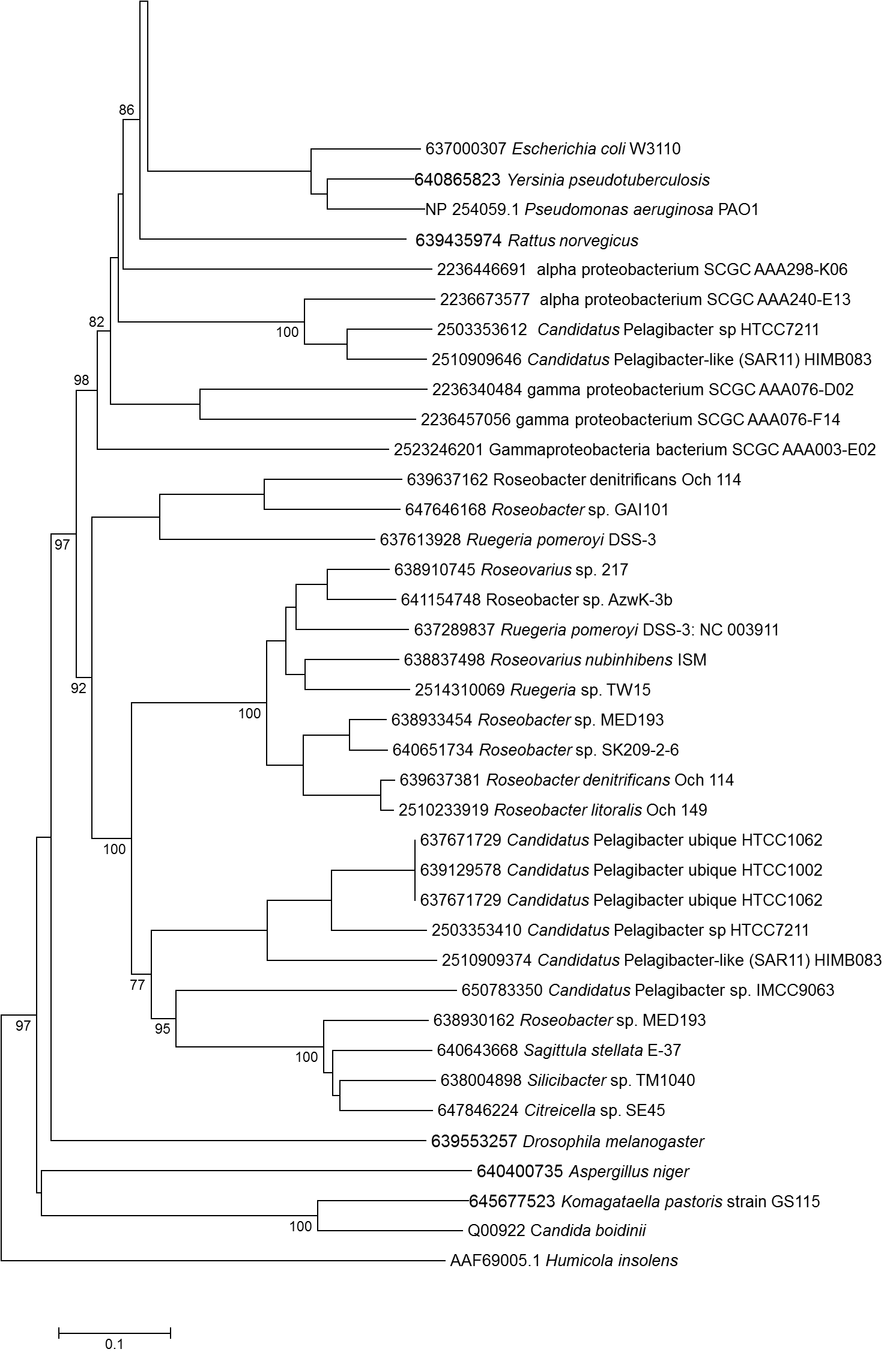


Un-characterised marine GMC oxioreductases

Characterised GMC oxioreductases

BetA

DddA

**Figure S4**


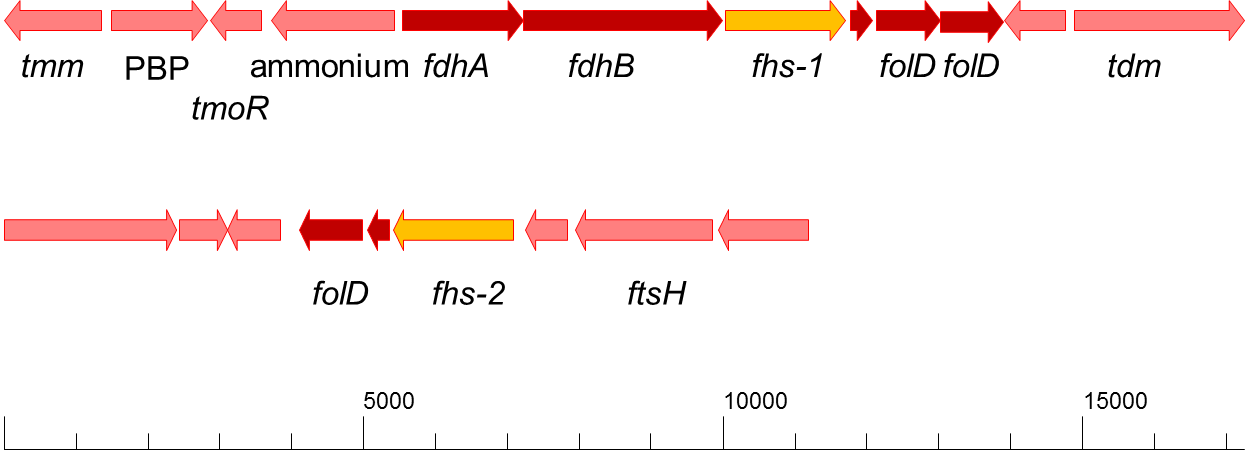


*amt*

**Figure S5**

H_4_F

OD_540_

OD_540_

OD_540_

**Figure S6**

H_4_F

**References**

Dennis, J.J., and Zylstra, G.J. (1998) Plasposons: modular Self-cloning minitransposon derivatives for rapid genetic analysis of gram-negative bacterial genomes. *Appl Environ Microbiol* **64**: 2710-2715.

Berntsson, R.P.A., Smits, S.H.J., Schmitt, L., Slotboom, D.-J., and Poolman, B. (2010) A structural classification of substrate-binding proteins. *FEBS Letters* **584**: 2606-2617.

González, J.M., Covert, J.S., Whitman, W.B., Henriksen, J.R., Mayer, F., Scharf, B. et al. (2003) *Silicibacter pomeroyi* sp. nov. and *Roseovarius nubinhibens* sp. nov., dimethylsulfoniopropionate-demethylating bacteria from marine environments. *Int J Syst Evol Micro* **53**: 1261-1269.

Kovach, M.E., Elzer, P.H., Steven Hill, D., Robertson, G.T., Farris, M.A., Roop Ii, R.M., and Peterson, K.M. (1995) Four new derivatives of the broad-host-range cloning vector pBBR1MCS, carrying different antibiotic-resistance cassettes. *Gene* **166**: 175-176.

Schäfer, A., Tauch, A., Jäger, W., Kalinowski, J., Thierbach, G., and Pühler, A. (1994) Small mobilizable multi-purpose cloning vectors derived from the *Escherichia* *coli* plasmids pK18 and pK19: selection of defined deletions in the chromosome of *Corynebacterium glutamicum*. *Gene* **145**: 69-73.
